# Low Temperature Impacts Root Physiological Characteristics and Related Microbial Community Diversity in the Rhizosphere of Japonica Rice

**DOI:** 10.3390/microorganisms14030632

**Published:** 2026-03-11

**Authors:** Zhenyu Liu, Yan Jia, Weibin Gong, Jian Jin, Shenyan Fu, Zhijie Luo, Wenhua Zhou, Jingguo Wang, Hongwei Zhao

**Affiliations:** 1 Key Laboratory of Germplasm Enhancement, Physiology and Ecology of Food Crops in Cold Region, Ministry of Education, Northeast Agriculture University, Harbin 150030, China; zhenyuliu666888@163.com (Z.L.); g314159261201@163.com (W.G.); fushenyan0713@163.com (S.F.); lzj03040304@163.com (Z.L.); zhouwenhua0502@163.com (W.Z.); 55190292@163.com (J.W.); 2 La Trobe Institute for Sustainable Agriculture and Food, Department of Ecology, Plant and Animal Sciences, La Trobe University, Melbourne Campus, Bundoora, VIC 3086, Australia; j.jin@latrobe.edu.au; 3 National Key Laboratory of Smart Farm Technologies and Systems, Harbin 150030, China

**Keywords:** *Oryza sativa* var. *japonica*, low-temperature treatment, root morphology and physiology, rhizosphere soil biodiversity

## Abstract

Low-temperature stress profoundly impairs rice root physiology and reshapes rhizosphere microbial communities. This 2023–2024 study examined its effects on *Oryza sativa* var. *japonica* across key growth stages. All treatments significantly suppressed root morphology and function, with the greatest reductions under combined tillering–booting stress (T3), followed by tillering (T1) and booting (T2). Strain DN428 exhibited a stronger cold tolerance than SJ10, with milder declines in root traits. Low-temperature stress elevated soil organic matter and total nitrogen while decreasing available phosphorus and potassium, leading to notable shifts in the microbial community structure and metabolic pathways. Weighted Gene Co-expression Network Analysis identified *lacZ*, *fucK*, and *rafA* in the MEbrown module as potential regulators of varietal cold responses. Mechanistically, yield loss in DN428 was mainly linked to the suppression of microbial gene expression, while in SJ10 it was associated with broader declines in microbial diversity and functional potential. Both varieties experienced yield reductions, accompanied by decreased root activity and nitrogen uptake. These findings underscore the necessity of a “gene–microbe–function” strategy to enhance microbial metabolism and optimize root–soil interactions under cold stress.

## 1. Introduction

Rice (*Oryza sativa* L.) is one of the most important staple crops globally, providing nourishment for approximately 56% of the world’s population [1]. Rice cultivation spans diverse climatic zones—from tropical and subtropical to temperate and even cooler regions—due to its sensitivity to specific temperature adaptability [2]. Its growth depends on favorable meteorological conditions (e.g., adequate water and light) and is particularly sensitive to temperature fluctuations [3,4]. Among various environmental stresses, chilling injury is one of the most detrimental to rice production [5,6]. Under ongoing climate change, extreme low-temperature events are expected to persist during rice growth and development [7,8]. In high-altitude regions, rice remains vulnerable to low temperatures throughout its life cycle, with the reproductive stage being critical due to its profound impacts on grain yield and quality [9,10,11,12,13]. Agricultural production in mid- to high-latitude regions has been severely affected by intensified climate variability, as vegetation in these areas is highly sensitive to temperature fluctuations [14,15,16].

Low-temperature stress is a major abiotic constraint on rice production, profoundly influencing both yield performance and geographical distribution [17,18]. Studies have shown that cold exposure impairs plant growth and root vigor, ultimately reducing biomass and yield [19,20,21,22,23]. However, most existing research focuses on the effects of low-temperature stress during a particular stage on rice yield. Several research studies have emphasized the importance of the vegetative (tillering) stage, where low temperatures delay development [24,25], impair nutrient uptake and root vigor [26,27], and reduce yield primarily by inhibiting tiller initiation and formation [28,29,30,31]. Other studies highlight the reproductive (booting/flowering) stage as particularly stressful [32,33], where chilling reduces pollen viability and increases empty spikelet rates, reflecting a rising trend of obstructive cold damage during tasseling and anthesis [34]. A critical knowledge gap remains: the combined effects of low-temperature stress across both tillering and booting stages are poorly understood. This study addresses this gap by systematically investigating the singular and compounded impacts of low-temperature stress at these key stages, aiming to clarify the mechanisms underlying cold damage in rice.

Crop yield arises from complex physiological and ecological processes driven by coordinated above- and below-ground interactions [35]. The root system—often termed the plant’s “brain” and “engine”—plays an indispensable role: it anchors the plant, absorbs water and mineral nutrients, and synthesizes plant hormones and signaling molecules that regulate above-ground growth and development [36]. Root activity, root length and the active absorption surface area are important physiological and morphological indexes of roots [37,38,39,40], which are closely related to yield formation [41,42]. For thermophilic crops like rice, low-temperature stress is a major factor limiting geographical distribution and yield [43]. It affects root morphology, structure, and function through multiple mechanistic pathways: morphologically, it suppresses root elongation, reduces root biomass, decreases lateral root and root hair density, and alters root architecture, weakening anchorage and soil resource exploration [44]; physiologically, it disrupts membrane fluidity, inhibits enzyme activity and cytoskeletal dynamics, impairs ion transporters (e.g., NH_4_^+^ transporters, aquaporins) critical for nutrient/water uptake, and disturbs reactive oxygen species homeostasis [45,46]. Even after temperature recovery, root morphological indices remain significantly lower than controls, with differences exacerbated by prolonged cold exposure [47].

A well-aerated, structurally loose, and nutritionally balanced rhizosphere environment promotes root elongation, branching, and vigor, enhancing water/nutrient absorption and supporting above-ground growth and yield [48]. Rhizosphere microorganisms are pivotal for soil–plant interactions: beneficial taxa (e.g., nitrogen-fixing bacteria, phosphorus-solubilizing microbes, plant growth-promoting rhizobacteria) directly enhance root growth and plant health via nitrogen fixation, the mobilization of soil-bound nutrients, the secretion of hormones (e.g., indole-3-acetic acid, IAA), and antagonism against pathogens [49]. They also indirectly improve soil nutrient availability by decomposing organic matter and facilitating nutrient cycling [50]. Temperature fluctuations alter these microbial processes: prolonged cold reduces soil nitrogen availability (NH_4_^+^, NO_3_^−^) and intensifies acidification [51], while cold-adapted phyla (*Actinobacteria*, *Acidobacteria*, *Chloroflexota*) maintain activity via membrane lipid remodeling, antifreeze protein synthesis, and extracellular polysaccharide production [52]. Other abiotic stresses (e.g., drought) also reshape microbial communities by modifying rhizosphere physicochemical properties [53].

Notably, shotgun metagenomic studies and functional gene analyses have revealed that microbial functional genes mediate plant stress responses [54,55], while network analysis (e.g., WGCNA [56,57]) and structural equation modeling identify key microbial functional modules linked to plant traits [58,59]. However, few studies integrate root morphological/physiological traits with rhizosphere microbial functional genomics under sequential low-temperature stress in *O. sativa* var. *japonica*. The “gene–microbe–function” linkage refers to a regulatory cascade in which stress-induced alterations in root exudates promote the enrichment of specific microbial taxa. The subsequent expression of functional genes within these taxa modulates root physiological responses, ultimately affecting plant performance. This conceptual framework remains insufficiently supported by empirical evidence in the existing literature.

To address these knowledge gaps, the following hypotheses were formulated:

**H1.**  *Compound low-temperature stress (applied at both tillering and booting stages) causes more severe impairment of root morphological traits and physiological traits than single-stage stress, with differential responses between cold-tolerant and cold-sensitive O. sativa var. japonica cultivars.*

**H2.**  *Low-temperature stress reshapes the rhizosphere microbial community structure, selectively enriching cold-adapted taxa and upregulating functional genes involved in nutrient cycling and phytohormone synthesis; the abundance of these genes is significantly correlated with root physiological traits.*

**H3.**  *Weighted Gene Co-expression Network Analysis (WGCNA) will identify key functional genes and microbial taxa that constitute a “gene–microbe–function” regulatory network mediating root stress responses and yield loss in O. sativa var. japonica.*

This study aims to: (1) investigate the regulatory effects of low-temperature stress at tillering (T1), booting (T2), and combined stages (T3) on the root traits of *O. sativa* var. *japonica* with differing cold tolerance; (2) elucidate concurrent changes in rhizosphere soil properties and the microbial community structure under these stresses; (3) identify key cold-responsive microbial functional genes using WGCNA; (4) propose an integrated “gene–microbe–function” model explaining variety-dependent yield loss mechanisms. The findings will advance the understanding of root–microbe interactions under cold stress and inform strategies to enhance *O. sativa* var. *japonica* cold tolerance.

## 2. Materials and Methods

### 2.1. Plant Materials and Growing Conditions

Two rice genotypes (DN428 and SJ10) were used in this study. DN428 exhibits a moderate cold tolerance, whereas SJ10 is characterized by a low cold tolerance [60]. The experiment was carried out at the Acheng Experimental Practice Base of Northeast Agricultural University, Harbin, Heilongjiang Province, China (45°44′–46°38′ N, 126°15′–127°30′ E), from April to early September in 2023 and 2024. The site is situated in a cold-temperate climatic zone with an average annual effective accumulated temperature exceeding 2700 °C.

### 2.2. Experimental Design

The experiment followed a split-plot design, in which the main-plot factor was low-temperature treatment, arranged in a randomized complete block, and the subplot factor was cultivar, randomly assigned within each main plot for three replicates; each subplot covered an area of 90 m^2^. The ambient temperature for watering served as the control. Low temperatures were administered at the following growth stages: T1, the tillering stage (initiated 7 days after regreening, cold water irrigation for 7 days); T2, the booting stage (when panicles reached approximately 1 cm, cold water irrigation for 7 days)); and T3, a combination of both stages. Details of the experimental layout are provided in the Supplementary Materials (Table S1). Nitrogen fertilizer was applied as urea (N 46.6%, 150 kg N ha^−1^), phosphorus as diammonium phosphate (120 kg ha^−1^), and potassium as potassium sulfate (100 kg ha^−1^). Nitrogen was applied as basal, tillering, and panicle fertilizers at a ratio of 6:3:1; phosphorus was applied entirely as a basal fertilizer; and potassium was applied at a ratio of 8:0:2. The same plots were used for low-temperature treatments in both 2023 and 2024 to ensure consistency. To address potential soil and microbial legacy effects, the year (Y) was treated as a random factor in the statistical model (see Section 2.5). Additionally, field management between cropping seasons included winter fallow and standard soil preparation to minimize carryover effects. To prevent interference between temperature treatments, adjacent plots were separated by 1.5 m wide buffer strips and independent irrigation channels. Each subplot was individually irrigated, and the water level was carefully monitored to ensure that cold-water irrigation was confined to the intended plots. Seeds were sown on 12 April and transplanted on 27 May in 2023, and sown on 8 April and transplanted on 21 May in 2024. Cold-water irrigation was maintained continuously (24 h day^−1^) from the onset of treatment. During the low-temperature treatments, an intelligent temperature-control system (XMT-908; Yuyao Gongyi Meter Co., Ltd., Yuyao, Zhejiang, China) was used to maintain water temperature at 15 ± 0.5 °C for the tillering-stage treatment and 17 ± 0.5 °C for the booting-stage treatment. Normal irrigation resumed after treatment, and all other field management practices followed standard agronomic guidelines.

### 2.3. Field Sampling and Lab Analyses

#### 2.3.1. Root Morphological and Physiological Traits

For each replicate, root samples were collected on the final day of low-temperature treatment, with three repetitions per treatment. Four plants with similar growth vigor were selected for each replicate. Whole plants were excavated using the full-sampling method [61], using the net bag method to collect root systems [62]. Prior to transplanting, insert an iron pipe with an internal diameter of 11 cm into the soil to a depth of 30 cm at each designated planting location. Remove the soil contained within the pipe to create a defined planting cavity. Subsequently, wrap a 10 cm diameter iron pipe with a nylon mesh bag and attach it to the inner wall of the larger pipe. Refill the inner pipe with soil, and then gradually extract both pipes in sequence. During harvest, excavate the root system together with the mesh bag, gently wash off the surrounding soil, and obtain an intact root structure for further analysis. Half of the collected roots were stored in a −4 °C ice box and used to determine root oxidation activity on the same day, while the remaining roots were stored at −20 °C for the determination of root morphological parameters. Root oxidation capacity was determined using the α-naphthylamine method described by Ramasamy et al. [63]. Root morphological traits, including total root length, surface area, and volume, were quantified using an LA-S Plant Root Analysis System (Wanshen, Hangzhou, China).

Xylem sap rate samples were collected on the final day of low-temperature treatment. Three rice plants with uniform growth were selected from each replicate, and sampling was performed in triplicate. At 19:00, the stems were cut 10 cm above the soil surface, and absorbent cotton was placed on each cut end, which was then covered with polyethylene film to collect exuded xylem sap. The cotton pads containing xylem sap were collected at 07:00 the following morning, and sap bleeding intensity was calculated based on the increase in cotton weight [64,65,66].

#### 2.3.2. Root Nitrogen Accumulation, Biomass and Yield

At the tillering, jointing, meiosis, full heading, grain filling, milky, dough, and maturity stages, five sampling points were selected, and measurements were conducted in triplicate. The plants were blanched at 105 °C for 30 min to inactivate enzymes, and then oven-dried at 80 °C to a constant weight before weighing, to measure the biomass. Ten sampling sites were used for seed testing, with three replications, to determine yield components. For each treatment, the actual grain yield was measured from a 2 m^2^ plot. Nitrogen content in the root system was determined using a CN analyzer (model: Skalar Primacs SN100-2C; Skalar Analytical, Breda, The Netherlands).

#### 2.3.3. Soil Sample Collection

For four time points, rhizosphere soil samples were collected: immediately following the conclusion of the low-temperature treatment during the tillering stage, with three replicates per treatment across both varieties, yielding a total of 12 samples; during the meiosis stage, with three replicates per treatment across both varieties, yielding a total of 18 samples; during the full heading stage, with three replicates per treatment for each variety, totaling 24 samples; and during the maturity stage, with three replicates per treatment for each variety, totaling 24 samples, resulting in a total of 78 samples. The specific procedure was as follows: rice roots were removed from the field plot, with surface soil removed to retain only a 0–2 mm layer of soil adhering closely to the roots. The roots were washed twice with PBS (phosphate-buffered saline) solution. The eluted soil suspension was transferred to a 50 mL sterile centrifuge tube, centrifuged at 6000× *g* for 20 min, and the supernatant and roots discarded [67]. The pelleted soil was thoroughly mixed. One portion was air-dried for physical and chemical index detection and analysis, and the other was stored at −80 °C for metagenomic sequencing analysis.

#### 2.3.4. Soil pH and Nutrient Contents

Soil pH was measured with a pH meter in a suspension with a soil-to-water ratio of 1:2.5 [68]. Soil organic matter (SOM) was determined using the K_2_Cr_2_O_7_-H_2_SO_4_ oxidation method [69]. Total nitrogen (TN) and total phosphorus (TP) were measured through the acid digestion method [70]. Available phosphorus (AP) in soil was measured using the antimony–molybdenum colorimetric method [71]. Available potassium (AK) in soil was measured using flame photometry (FP-6410; Xinyi Instruments, Shanghai, China) [72].

#### 2.3.5. Soil Metagenomic Sequencing

DNA extraction, library construction, and metagenomic sequencing

Total genomic DNA was extracted from 78 samples obtained at the aforementioned four time points using the FastPure Soil DNA Isolation Kit (Magnetic bead) (MJYH, Shanghai, China). Concentration and purity of the extracted DNA were determined with TBS-380 and NanoDrop2000 (Shanghai Meiji Biomedical Technology Co., Ltd., Shanghai, China), respectively. DNA extract quality was checked on 1% agarose gel.

DNA extract was fragmented to an average size of about 400 bp using Covaris M220 (Gene Company Limited, Shanghai, China) for paired-end library construction. The average library molar concentration was 10.5 nM. A paired-end library was constructed using NEXTFLEX^®^ Rapid DNA-Seq (Bioo Scientific, Austin, TX, USA). Adapters containing the full complement of sequencing primer hybridization sites were ligated to the blunt-end of fragments. Paired-end sequencing was performed on Illumina (Illumina, San Diego, CA, USA) at Majorbio Bio-Pharm Technology Co., Ltd. (Shanghai, China) using NovaSeq 6000 (Shanghai Meiji Biomedical Technology Co., Ltd., Shanghai, China). The sequencing method was PE, and the read length was 150 bp.

Sequence quality control and genome assembly

The data were analyzed on the free online platform of Majorbio Cloud Platform (www.majorbio.com). Briefly, the paired-end Illumina reads were trimmed of adaptors, and low-quality reads (length < 50 bp or with a quality value < 20 or having N bases) were removed by fastp [73] (https://github.com/OpenGene/fastp, accessed on 1 July 2024, version 0.20.0). The average clean data of the samples were 11 Gb. Host-derived reads were filtered by mapping to the Oryza sativa reference genome (IRGSP-1.0) using Bowtie2 (v2.4.5) with default parameters. Clean reads from all samples were co-assembled using MEGAHIT (v1.2.9) with default parameters. Contigs longer than 300 bp were retained for subsequent analysis. Assembly metrics included an N50 of 2147 bp and a maximum contig length of 98,532 bp.

Gene prediction, taxonomy, and functional annotation

Open reading frames (ORFs) from each assembled contig were predicted using MetaGene [74] (http://metagene.cb.k.u-tokyo.ac.jp/, accessed on 1 July 2024). Only ORFs with a length ≥ 100 bp were retained. The predicted ORFs were translated into amino acid sequences using the NCBI translation table.

Construction of a non-redundant gene set

A non-redundant gene catalog was constructed using CD-HIT [75] with parameters set at 90% sequence identity and 90% coverage (-c 0.9 -aS 0.9). High-quality reads were mapped back to this catalog using SOAPaligner (soap2.21) with 95% identity to calculate gene abundance.

Species and function notes

Representative sequences of the non-redundant gene catalog were aligned against the NCBI NR database (version: 2023-11-26) using Diamond [76] (v0.8.35) with an e-value cutoff of 1 × 10^−5^. Taxonomic assignment was performed based on the lowest common ancestor algorithm. For functional annotation, sequences were also aligned against the Kyoto Encyclopedia of Genes and Genomes (KEGG) database (Release 107.0) using Diamond with the same e-value threshold. Only the best hits with an e-value ≤ 1 × 10^−5^ and bit-score ≥ 60 were retained for pathway mapping.

Microbial diversity and statistical analysis

Alpha diversity indices (Shannon, Chao1, and Simpson) were calculated based on gene abundance profiles using the vegan package (v2.6-4) in R. Beta diversity was assessed using Bray–Curtis dissimilarity and visualized via principal coordinate analysis (PCoA). Permutational multivariate analysis of variance (PERMANOVA) with 999 permutations was applied to test the effects of treatment, variety, and growth stage.

Weighted Gene Co-expression Network Analysis (WGCNA) was performed using the WGCNA package (v1.72) in R. A soft-thresholding power of 12 was selected based on scale-free topology criterion (R^2^ > 0.85). Modules were detected using dynamic tree cutting with a minimum module size of 30 genes. Module–trait associations were assessed using Pearson correlation, with *p*-values adjusted using the false discovery rate (FDR) method.

Structural Equation Modeling (SEM) was conducted using the lavaan package (v0.6-16) in R. Model fit was evaluated using the chi-square test (χ^2^/df < 3), comparative fit index (CFI > 0.90), root mean square error of approximation (RMSEA < 0.08), and standardized root mean square residual (SRMR < 0.08).

### 2.4. Calculation of Cold-Response Index

Cold-response index $\left(CRI,\%\right)=\frac{T}{C}\times 100$, where *T* represents the physiological parameters under stress, and *C* is the control.

### 2.5. Statistical Analysis

To account for potential interannual variation and plot carryover effects, the year (Y) was treated as a random factor in the statistical model, while variety (V) and treatment (T) were considered fixed factors. Analysis of variance (ANOVA) was applied using the SPSS 26.0 software package (IBM SPSS Statistics 26, Armonk, NY, USA). The statistical model included sources of variation due to year (Y), variety (V), treatment (T), and Y × V, Y × T, V × T, and Y × V × T interactions. Least significant difference (LSD) was used to estimate the difference between the two years for an identical variety, and between the two varieties under an identical year condition [77]. Graphs were generated with Origin 20.0, and the standard errors of the means were calculated and represented in the graphs as error bars. Linear regression analysis was performed with SPSS 26.0. For multivariate and network-based analyses, including Weighted Gene Co-expression Network Analysis (WGCNA) and Structural Equation Modeling (SEM), detailed methodologies are provided in Section 2.3.5.

## 3. Results

### 3.1. Root Morphological and Physiological Traits and Root Nitrogen Accumulation and Biomass

**Figure 1:**
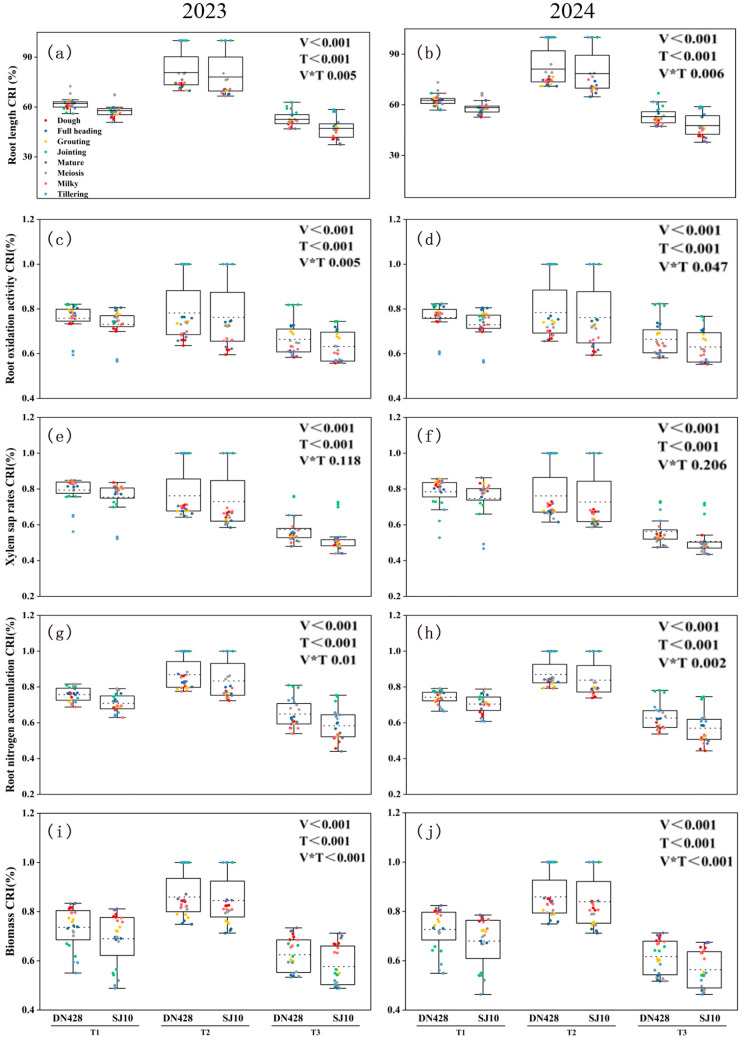
Box plots showing the cold-reaction index (CRI) of total root length (**a**,**b**), root oxidation activity (**c**,**d**), xylem sap rates (**e**,**f**), root nitrogen accumulation (**g**,**h**) and biomass (**i**,**j**) of rice varieties Songjing 10 (SJ10) and Dongnong 428 (DN428) under low-temperature treatment at the tillering and booting stage during 2023 and 2024. The significant levels of variety (V) and temperature (T) effects and their interactions are also shown. Data from different growth stages are pooled.

Low-temperature treatment significantly reduced root growth and physiological activity in both rice varieties (Figure 1). A three-way ANOVA (Year × Variety × Treatment) revealed that the main effects of variety (V) and treatment (T) on all measured root traits were highly significant (*p* < 0.01), while the year (Y) effect and most interactions were not significant (detailed statistics in Table S2). Consequently, the cold-response index (CRI, %) of root length, root oxidation activity, xylem sap rate, root nitrogen content, and biomass varied significantly among varieties and treatments, but not between years (the trends of changes in the root surface area and root volume were similar to root length; see Figure S1). Compared with the control, the T3 treatment showed the strongest inhibitory effect, followed by T1 and T2. SJ10 exhibited a consistently greater sensitivity to low-temperature treatment than DN428, with larger reductions in all root traits. For instance, under T3 treatment, the decreases in CRI (% of control) for root length, root oxidation activity, xylem sap rate, root nitrogen accumulation, and biomass in SJ10 were 3.6–18.0%, 2.4–8.1%, 5.1–18.1%, 5.1–17.7%, and 3.9–15.8% lower, respectively, than those observed in DN428 (all *p* < 0.05; Figure 1).

### 3.2. Effects of Low-Temperature Treatment on Yield and Yield Components of Rice

Yield and its components were significantly influenced by treatment and variety, but not by year. Additionally, the interaction between variety and treatment was significant or highly significant, except for the 1000-grain weight (Table 1). Compared to the control, the number of effective panicles in both DN428 and SJ10 exhibited no significant differences under T2 treatment but declined significantly under T1 and T3. The 1000-grain weight decreased under T2 and T3 but not under T1. Grains per panicle, seed setting rate, and yield all decreased significantly under low-temperature treatments, with the magnitude of reduction following T3 > T2 > T1. SJ10 exhibited a greater sensitivity, with yield reductions 4.0–5.3% larger than those in DN428.

### 3.3. Effects of Low-Temperature Treatment on Soil pH and Nutrient Contents

**Figure 2:**
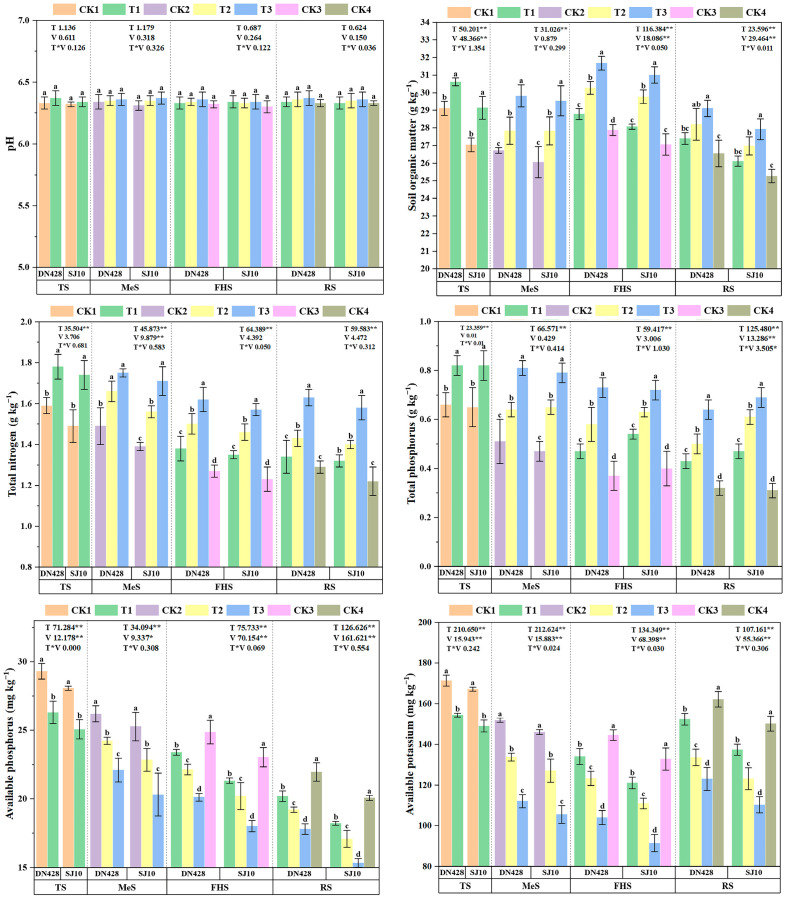
Effect of low-temperature treatment on soil pH and nutrient contents of rice rhizosphere soil in cold region during tiller and booting stage. CK1–CK4 are the corresponding control group data for the tillering stage, meiosis stage, full heading stage and mature stage, respectively. The significant levels of variety (V) and treatment (T) effects and their interactions are also shown. Values with a column followed by different letters are significantly different at *p* < 0.05. * Significant at *p* < 0.05; ** Significant at *p* < 0.01.

Compared with the control, the SOM, TN and TP contents in the rhizosphere soil of DN428 and SJ10 increased under low-temperature treatment; AP and AK decreased significantly, whereas the pH showed no significant differences across all the growth stages. The magnitude of these changes was treatment-dependent, with the order T3 > T2 > T1. At maturity, for instance, TN in DN428 increased significantly only under T2 and T3 treatments, whereas SJ10 exhibited significant increases under all treatments. Notably, compared to DN428, SJ10 showed greater reductions in TP (1.5–3.1%), SOM (4.1–4.7%), AK (7.8–10.4%), and AP (9.8–13.8%) at maturity, respectively (Figure 2).

### 3.4. Metagenomic Analysis of Rice Rhizosphere Microbial Under Low-Temperature Treatment

#### 3.4.1. Soil Alpha Diversity

Alpha diversity is mainly used to study the diversity of communities within a certain lifetime (or within a sample). It can obtain information such as the richness and diversity of species/functions in the environmental community by evaluating a series of Alpha diversity indices. The Alpha diversity of rhizosphere microbial communities responded to low-temperature treatment in a stage-specific manner (Table 2). Community richness (ACE index) remained relatively stable across all growth stages (37,000–39,000). In contrast, community diversity (Shannon and Simpson indices) was most vulnerable during the full heading stage, showing significant reductions under stress (e.g., Shannon index in SJ10-T3 decreased by ~15% compared to control, *p* < 0.05).

At the tillering stage, varietal differences were prominent, with SJ10 showing a significant increase in ACE under T1 (*p* < 0.05), a response absent in DN428. During the meiosis stage, treatment effects were minimal, though SJ10 exhibited opposing trends between Shannon and Simpson indices. The full heading stage was the most responsive, with both Shannon and Simpson indices varying significantly among treatments (*p* < 0.01) and the Shannon index also differing between varieties (*p* < 0.05). By maturity, the treatment effects on Alpha diversity indices were no longer significant (Table 2).

#### 3.4.2. Soil Beta Diversity

**Figure 3:**
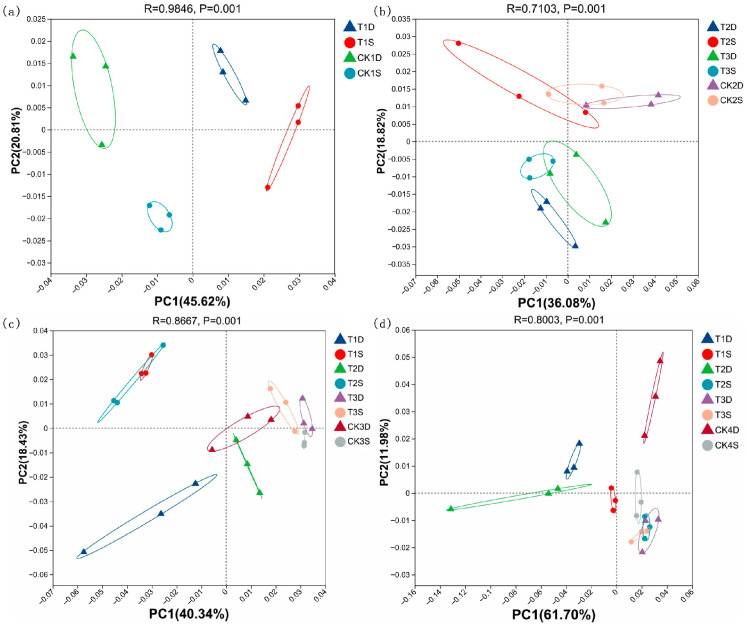
PCoA analysis of rhizosphere soil microorganisms at tillering stage (**a**), meiosis (**b**), full heading (**c**) and maturity stages (**d**) at the phylum level under low-temperature treatment at the tillering and booting stages. D is DN428; S is SJ10. CK1–CK4 are the corresponding control group data for the tillering stage, meiosis stage, full heading stage and mature stage, respectively.

The Beta diversity analysis revealed a significant separation between treatments and the control, especially under T3 (Figure 3). Distinct shifts in the microbial community composition were observed across all growth stages. At the tillering and meiosis stages, significant differences were detected between the DN428 and SJ10 treatments (T1–T3) compared with the control (Figure 3a,b). At the full heading stage, no significant difference in microbial composition was found between DN428 under T2 and the control; however, the T1 and T3 treatments caused significant deviations. For SJ10, there was no significant difference between T1 and T2, but both differed from T3 (Figure 3c). At maturity, all treatments altered the microbial composition relative to the control in both varieties, with no difference between the T2 and T3 treatment in SJ10 (Figure 3d).

#### 3.4.3. Microbial Species Composition

At the phylum level, *Anaerolinea* dominated across all growth stages samples; however, the relative proportions of these taxa varied. Furthermore, the relative abundance of several key taxa varied under treatments (Figure S2). During the tillering stage, the *Arthrobacter* abundance significantly decreased under T1 in both varieties compared to the control (*p* < 0.01; Figure S3a). For *Gemmatimonas*, a significant reduction in abundance was observed under the T2 treatment in both varieties, but under T3 a significant decrease was observed only in SJ10 (Figure S3b). At the full heading stage, the relative abundance of *Arthrobacter* in SJ10 decreased significantly under T1 and T2 but not T3, relative to the control. In DN428, a significant decrease was observed under T1 (Figure S3c). During the mature stage, the relative abundance of *Labilithrix* in DN428 was lower under T1 and T2 treatments than under T3, but neither differed from the control (Figure S3d).

#### 3.4.4. Linking Microbial Features to Plant and Soil Parameters

**Figure 4:**
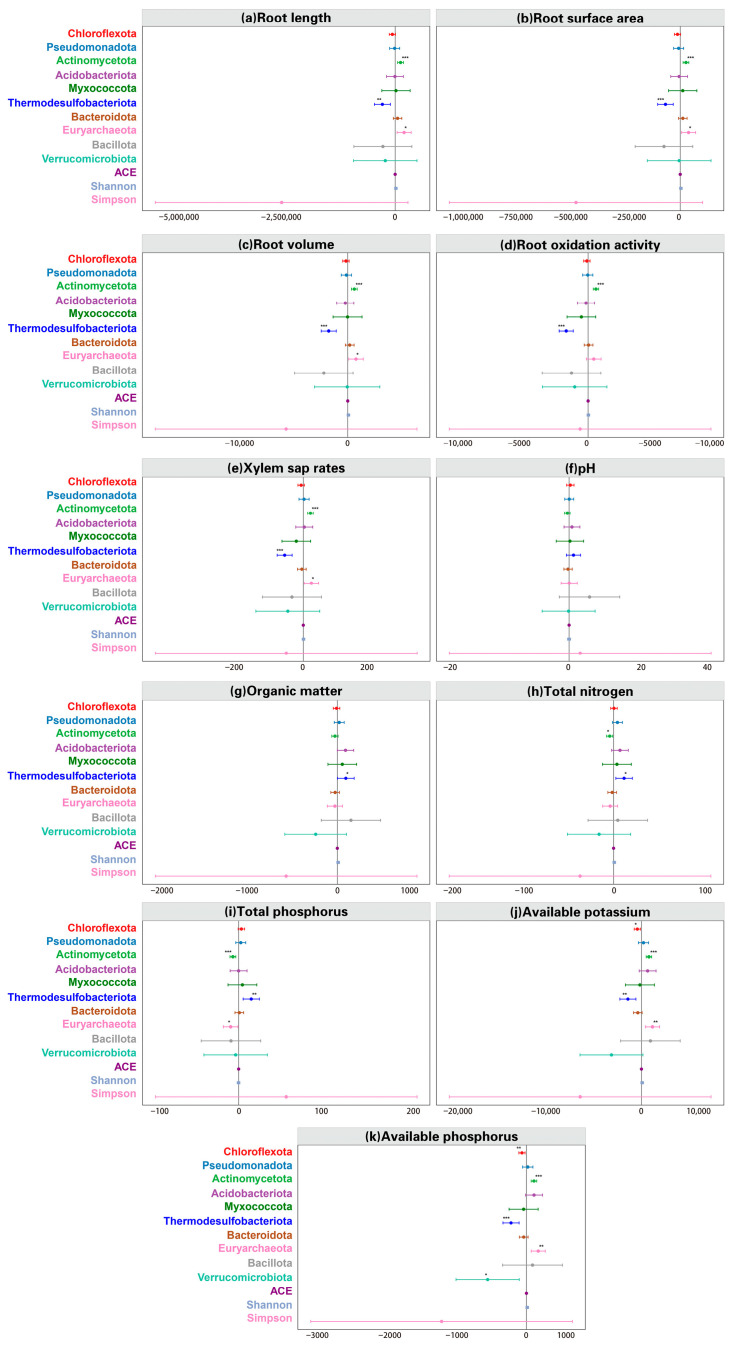
Mixed-effects models for soil microbial α-diversity and the relative abundance of the top ten microbial taxa at phylum level, in relation to rice root morphological and physiological traits and soil pH and nutrient contents. *p* value is displayed with asterisk. * represents *p* < 0.05, ** represents *p* < 0.01, *** represents *p* < 0.001.

Mixed-effects models were employed to identify specific microbial phyla significantly associated with root traits and soil nutrient statuses (Figure 4). The results indicated that *Actinomycetota* and *Euryarchaeota* were significantly associated with root length, root surface area, root volume and xylem sap rate (all *p* < 0.05, r > 0.40), although *Euryarchaeota*’s positive effect on root oxidation activity (*p* = 0.134) showed no significant differences (Table S3). In contrast, *Thermodesulfobacteriota* exhibited a significant negatively effect on root traits (*p* < 0.01). The Alpha diversity index ( Shannon, Simpson and ACE) had no significant associations with root morphological and physiological traits. *Actinomycetota* significantly suppressed TN and TP, while markedly enhancing AK and AP. Meanwhile, *Thermodesulfobacteriota* exhibited the opposite pattern. Additionally, *Chloroflexota* was found to significantly inhibit AK and AP.

Redundancy analysis (RDA) identified that AK and AP were the dominant factors shaping microbial community structure across all growth stages. At the tillering stage, AP and AK in the RDA biplot overlapped, suggesting a strong correlation (RDA1: 24.38%, RDA2: 17.68%) (Figure S4a). At the full heading stage, the arrows for SOM and TN coincided, indicating a close association (RDA1: 20.74%, RDA2: 7.04%) (Figure S4c).

The Spearman correlation coefficient was employed to assess variety- and treatment-specific associations (Figure S5, for full statistical details, see Table S4). In the DN428 treatment group, several phyla (e.g., *Pseudomonadota* and *Cyanobacteriota*) were positively correlated with TN and TP, while others showed specific links to AP or negative correlations with AK and SOM. The DN428 control group exhibited a broader spectrum of positive correlations, particularly linking *Acidobacteriota*, *Gemmatimonadota*, *Actinomycetota* and *Pseudomonadota* with AP and TP. In SJ10, both treatment and control groups were characterized by strong positive correlations of *Gemmatimonadota* with TN/TP and of *Candidatus Rokubacteria* with AP. Notably, *Verrucomicrobiota* was consistently negatively correlated with TN, TP and AP in the SJ10 control group.

#### 3.4.5. Predicted Functional Profiles of Microbial Communities

**Figure 5:**
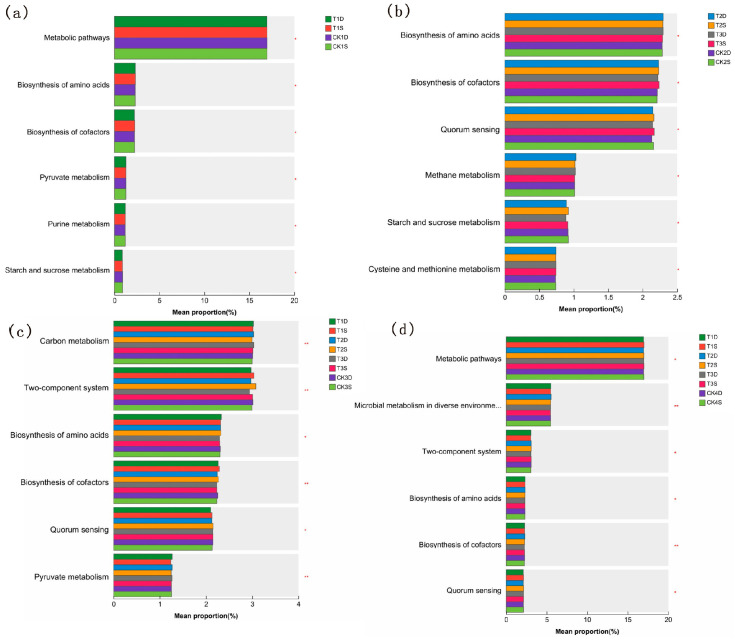
Rhizosphere soil microorganisms KEGG metabolic pathway at tillering stage (**a**), meiosis (**b**), full heading stage (**c**) and maturity stages (**d**) at the phylum level under low-temperature treatment at the tillering and booting stages. D is DN428; S is SJ10. CK1–CK4 are the corresponding control group data for the tillering stage, meiosis stage, full heading stage and mature stage, respectively. *p* value is displayed with asterisk. * represents *p* < 0.05, ** represents *p* < 0.01.

During the tillering and maturity stages, “Metabolic pathways” was the dominant metabolic pathway (Figure 5a,d). At the meiosis stage, pathways for “Biosynthesis of amino acids”, “Biosynthesis of cofactors” and “Quorum sensing” differed significantly between varieties (Figure 5b). At the full heading stage, “Carbon metabolism” and the “Two-component system” were key. Compared with the control, the relative abundance of the “Two-component system” pathway decreased in DN428 (by 1.3–2.3%) under low-temperature treatments, but increased in SJ10 (by 0.7–3.0%). The “Carbon metabolism” pathway increased slightly in DN428 (by 0.7–1.0%) under all treatments and in SJ10 under T1 and T3 (0.7%) (Figure 5c).

#### 3.4.6. Microbial Correlation Network Analysis

**Figure 6:**
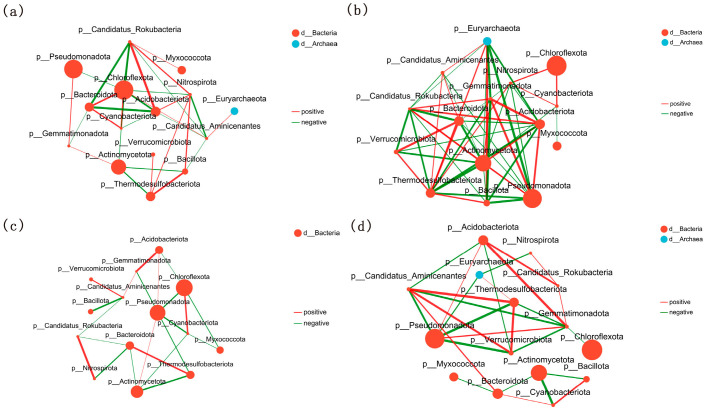
One-factor correlation network diagram between rhizosphere soil phylum level microorganisms and function in DN428 treatment group (**a**), DN428 control group (**b**), SJ10 treatment group (**c**) and SJ10 control group (**d**). The number of samples in each figure is n. (**a**) n = 27, (**b**) n = 12, (**c**) n = 27, (**d**) n = 12.

In the DN428 treatment group, 62 significant correlations were identified among the top 15 most abundant phyla. Notably, *Chloroflexota* and *Candidatus Rokubacteria* exhibited key connectors (Figure 6a). In the DN428 control group, 110 significant correlations were observed, with *Actinomycetota*, *Thermodesulfobacteriota*, *Pseudomonadota* and *Acidobacteriota* as major hubs (Figure 6b). The SJ10 treatment group had fewer connections (42) (Figure 6c) than the control (56) (Figure 6d), and the role of the connectors shifted. This indicates that low-temperature treatment strengthened the network role (e.g., increased degree centrality) of *Chloroflexota* but weakened that of *Pseudomonadota* and *Actinomycetota* (Figure S6).

### 3.5. WGCNA

#### 3.5.1. Modules and Hub Genes Associated with Root Traits

**Figure 7:**
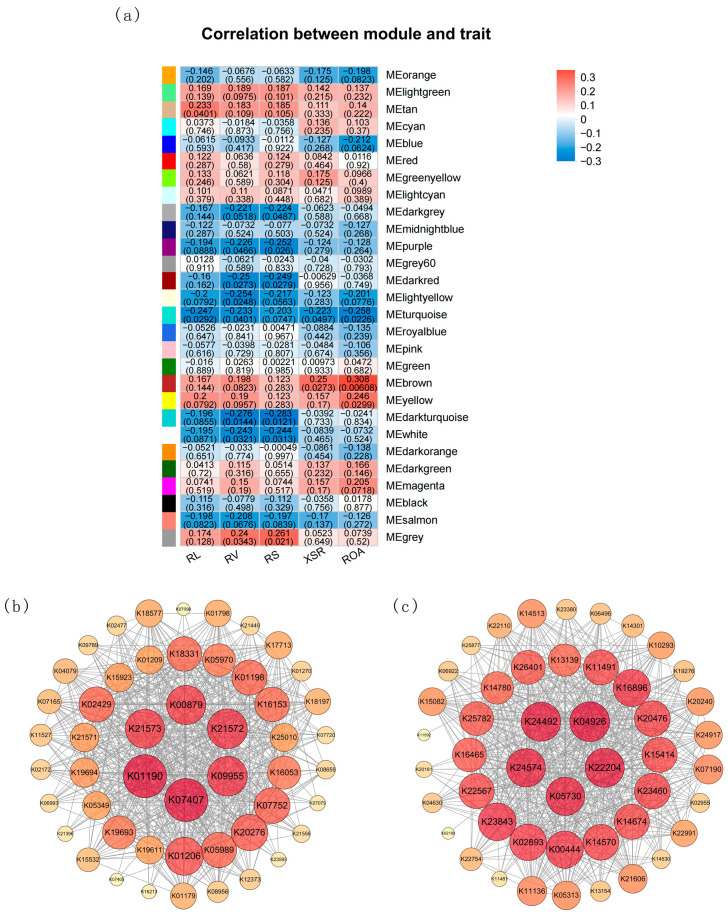
Correlation analysis between co-expression network module and root morphological and physiological traits (**a**) (RL: root length; RV: root volume; RS: root surface area; XSR: xylem sap rate; ROA: root oxidation activity). Regulatory network of key genes in the MEbrown module (**b**). Regulatory network of key genes in the MEdarkturquoise module (**c**).

Weighted Gene Co-expression Network Analysis (WGCNA) [78] was performed on metagenomic data and root traits and identified 28 gene modules (Figure S7). The correlation analysis revealed that the MEbrown module was moderately, positively associated with root oxidation activity (r = 0.308, *p* = 0.006). The MEdarkturquoise module was weakly (but statistically significantly) negatively associated with root surface area (r = −0.283, *p* = 0.012). Overall, root oxidation activity and root surface area showed the strongest associations with gene modules. Based on these results, KEGG Orthology (KO) terms from the MEbrown and MEdarkturquoise modules were selected for further analysis (Figure 7a).

In the MEbrown module, the hub genes included *susC* (K21573), *lacZ* (K01190), *fucK* (K00879), *susD* (K21572), *E3.2.1.22B, galA, rafA* (K07407), and K09955 (Figure 7b). In the MEdarkturquoise module, the key genes were *BEST* (K22204), *KCNQ1, KV7.1* (K04926), *LRRN1_2_3* (K24492), *TEX11* (K24574), and *VAV* (K05730) (Figure 7c). These genes are likely involved in regulating root development under low-temperature treatment.

#### 3.5.2. Modules and Hub Genes Associated with Soil Nutrients

Weighted Gene Co-expression Network Analysis (WGCNA) was conducted on genes with soil parameters and also yielded 28 modules (Figure S8). The MEblue module was strongly positively correlated with AP (r = 0.712, *p* = 0) and AK (r = 0.486, *p* < 0.001), while the MEgreen module was negatively correlated with AP (r = −0.724, *p* = 0) and AK (r = −0.395, *p* < 0.001) (Figure S9a). Based on a comprehensive evaluation, the MEblue and MEgreen modules were not only significantly associated with soil pH and nutrient contents but were also correlated with multiple parameters. Therefore, co-expressed genes within the MEblue and MEgreen modules were selected for further functional analysis.

Hub genes in the MEblue module (e.g., *dnaE2* (K14162), *lhr* (K03724), *ALDH* (K00128) and *ligD* (K01971)) (Figure S9b) and the MEgreen module (e.g., *wza, gfcE* (K01991), *atoC* (K07714), *cckA* (K13587), *TMTC* (K23424) and *zraS, hydH* (K07709)) (Figure S9c) were potentially involved in stress response and nutrient cycling regulation.

### 3.6. Integrated Pathways to Yield Loss Under Low-Temperature Treatments

**Figure 8:**
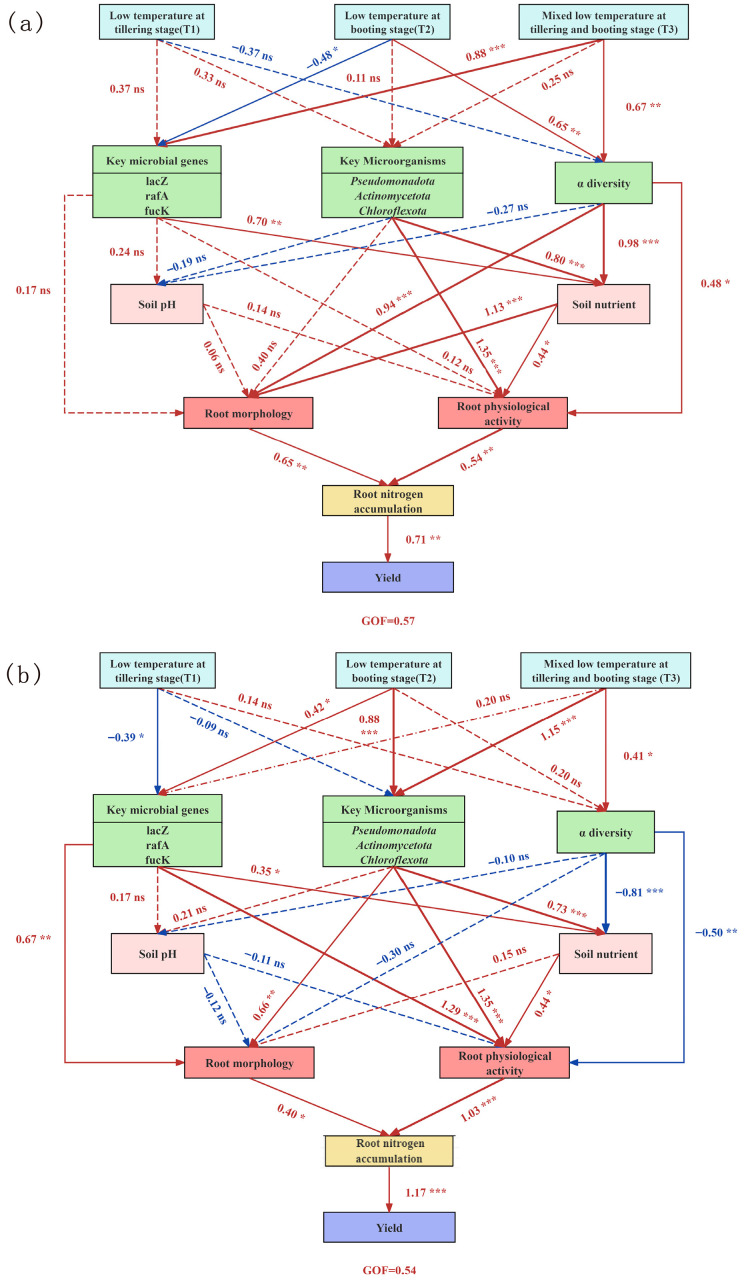
Structural equation modeling (SEM) between key environmental factors and rice growth and different low-temperature treatments. Red solid arrows denote significant positive effects, while red dashed arrows indicate positive but non-significant effects. Blue solid arrows denote significant negative effects, and blue dashed arrows indicate negative but non-significant effects. T1—low temperature at tillering stage; T2—low temperature at booting stage; T3—mixed low temperature at tillering and booting stage. (**a**) SEM of DN428. (**b**) SEM of SJ10. * Significant at *p* < 0.05; ** Significant at *p* < 0.01; *** Significant at *p* < 0.001.

Structural equation modeling (SEM) delineated variety-specific pathways (Figure 8). The overall model fit was satisfactory for both varieties (standardized root mean square residual, SRMR < 0.08). For DN428 (Figure 8a), the primary pathway was initiated by low-temperature treatments at the booting stage (T2) and the combined stage (T3). These treatments significantly influenced the α-diversity of the soil microbial community (path coefficient for T2 = 0.65, T3 = 0.67, *p* < 0.01), which in turn affected the soil organic matter (SOM) content (coefficient from “a diversity” to “Soil nutrient” = 0.98 *p* < 0.01). This cascade ultimately impaired root physiology and nitrogen accumulation, leading to yield loss.

For SJ10 (Figure 8b), the pathways were more complex and involved key microbial genes. The T1 treatment at the tillering stage exerted a significant direct negative effect on the expression of key microbial genes (e.g., *fucK*, *lacZ*, *rafA*) (path coefficient = −0.39, *p* < 0.05). In parallel, the T2 and T3 treatments markedly altered microbial community richness. These distinct pathways converged on a critical node: lowering soil available phosphorus (AP) availability, which subsequently strongly inhibited root activity (coefficient = 0.44, *p* < 0.05) and ultimately caused severe yield loss (coefficient from N accumulation to yield = 1.17, *p* < 0.01).

## 4. Discussion

### 4.1. Low Temperature Impairs Root Functionality and Limits Yield

Low temperatures significantly suppressed root morphological and physiological traits at both developmental stages, aligning with previous research outcomes [5]. However, varietal differences in cold tolerance were evident. Specifically, the cold-tolerant cultivar DN428 exhibited less reduction in root morphological and physiological traits compared to SJ10 under low-temperature treatment, indicating that DN428 maintained a superior root morphology and thus more efficiently transported water and nutrients to the aerial parts. Moreover, this study observed that, even in later growth stages, the root morphological and physiological traits of plants exposed to low temperatures remained lower than those under normal conditions. These results suggest that low-temperature treatment not only directly inhibits root growth and elongation during critical stages but also accelerates root senescence in subsequent stages, consistent with the findings of Yang [47]. This may be attributed to inhibited root development, delayed system growth, cellular dehydration, and diminished nutrient transport capacity. Ultimately, the reduced root transport function limits nutrient and water supply to the shoots, impairs photosynthesis, decreases carbohydrate return to roots, and weakens root respiration, collectively hastening the aging of roots. This cascade of effects underscores the long-term detrimental influence of low temperatures on root system development in cold-region *O. sativa* var. *japonica*.

Low-temperature treatment is a major constraint on rice yield, posing a significant barrier to achieving both high and stable production. Gunawardena et al. [79] reported that extreme low temperatures affecting rice panicles and roots during the booting stage led to a marked reduction in the seed-setting rate. Similarly, Jiang et al. [80] noted that exposure to natural cold and low-temperature treatment during the booting period resulted in a decrease in the 1000-grain weight. In this study, low-temperature treatment had varying impacts on different treatments. Compared to the control, the effective number of ears in T2 showed no significant differences, whereas T1 and T3 experienced substantial reductions. The effect of T1 on the 1000-grain weight was negligible; however, significant decreases were observed in T2 and T3. Additionally, low-temperature treatment led to declines in the number of grains per panicle, the seed-setting rate, and the theoretical yield of the cold-tolerant *O. sativa* var. *japonica* variety DN428, though the magnitude of the reduction was smaller than that observed in SJ10.

### 4.2. Low Temperature Alters the Rhizosphere Nutrient Availability

This study reveals that changes in the soil pH and nutrient content induced by low-temperature stress provide crucial soil environmental evidence for understanding the physiological and microbial response mechanisms specific to rice varieties. All treatment groups exhibited consistent patterns: significant increases in the SOM, TN, and TP content, alongside significant decreases in the AP and AK content. This indicates that the rhizosphere environment under low-temperature treatment is shifting from a mineralization-dominated state towards nutrient immobilization [81]. This shift likely stems from the inhibition of microbial activity and extracellular enzyme activity by low temperatures [82]. The reduced microbial activity in the rhizosphere delayed the decomposition of organic substrates, thereby hindering the conversion of soil nutrients into plant-available forms. This ultimately constrained nutrient availability during the critical growth stages of rice [83]. Furthermore, throughout the sampling period, both AK and AP emerged as key drivers of microbial community dynamics. This aligns with prior research concluding that ammonium nitrogen (AN) and AP constitute core drivers of variation in rhizosphere microbial communities [84,85].

Notably, this study observed low temperatures inducing TN accumulation in soil, whereas other long-term cold stress studies reported TN depletion [86]. This apparent contradiction likely stems from differing experimental durations and intensities: short-term or cyclical cold primarily inhibits microbial mineralization, leading to TN fixation in organic forms, whereas prolonged, sustained cold may completely suppress microbial biomass and nitrogen fixation, resulting in net TN consumption. Furthermore, rice cultivars differentially regulate soil nitrogen fixation and transformation processes through pathways such as altering root exudate composition. Initial soil physicochemical properties (e.g., texture, organic matter content) strongly mediate nutrient cycling responses under low temperatures [82]. This suggests that low temperatures influence soil nitrogen pools through the combined effects of microbial processes and plant–microbe interactions within specific environmental contexts.

The gradient responses observed across treatment groups (particularly pronounced under T3) correlate closely with progressively inhibited root morphophysiological traits. The significant accumulation of SOM and TN under T2 and T3 treatments likely reflects diminished root nutrient uptake capacity and weakened microbial metabolic activity, leading to the retention of organic compounds in the soil [87]. Conversely, the decline in AP and AK signaled emerging nutrient supply constraints [88], potentially exacerbating the observed physiological root suppression in both rice varieties. Crucially, the differential nutrient dynamics between rice varieties—particularly SJ10, exhibiting more pronounced reductions in AP, AK, and TP contents than DN428—supports the prior inferences regarding SJ10’s heightened sensitivity to cold-induced rhizosphere dysfunction. As suggested by the hypothesis-generating structural equation modeling results, alterations in soil nutrient status may have exacerbated the disruption of the microbial community structure in SJ10. In contrast, the DN428 cultivar maintained relatively high nutrient retention and availability, exhibiting a stress response primarily characterized by the downregulation of key microbial functional genes, without experiencing widespread microbial community collapse.

### 4.3. Microbial Community Composition and Functional Gene Regulated by Low Temperature

This study systematically investigated the effects of low-temperature treatment on rhizosphere soil microbial community diversity by subjecting rice plants to such treatment during the tillering and heading stages. The results indicated that, during the heading stage, both the ACE index and the Simpson index of rhizosphere microorganisms in the SJ10 variety were significantly lower under low-temperature treatment compared to the control. This suggests that low temperatures suppressed soil microbial metabolism and enzyme activity in the rhizosphere, thereby slowing the mineralization of TN and TP mineralization processes and their conversion into available nutrients (AP, AK), thereby creating a soil nutrient-limited environment. This further suppressed the growth of microbial groups dependent on high nutrient turnover rates, leading to reduced α-diversity in the rhizosphere microbial community [89]. In the study, the significant reduction in *Actinomycetota* abundance observed in both varieties under low-temperature treatment during the tillering stage may be directly linked to the suppression of their functional roles in degrading organic matter and converting recalcitrant compounds.

However, other studies indicate that, under abiotic stress, the composition of plant root exudates may alter, thereby specifically stimulating or selectively enriching certain microbial groups, ultimately increasing microbial community diversity [90]. This study observed that SJ10 exhibited a significantly higher ACE index than the control under low-temperature treatment during tillering, while DN428 showed a markedly elevated relative abundance of the *Pseudomonadota* phylum across all treatments at maturity. These findings precisely support this perspective. This suggests that the impact of low temperatures on microorganisms is not solely inhibitory but more likely represents a functional reshaping of the community based on environmental selection [86]. Notably, unlike general microbial stress responses, where metabolic genes are involved in basic adaptation, this study identifies that *lacZ*, *rafA*, and *fucK* in the rice rhizosphere specifically mediate the coupling of microbial carbon metabolism and plant–microbe nutrient exchange—facilitating the microbial utilization of root exudate-derived carbon while sustaining energy supply (via NADPH production) for both microbial stress resistance and root physiological function. This rhizosphere-specific functional association distinguishes the findings presented here from broader microbial stress response reports, highlighting their relevance to rice-specific cold adaptation. Rice varieties with differing cold tolerance may actively recruit beneficial microorganisms to assist in stress resistance by regulating root exudates. This reshaping process centers on microbial functional traits rather than mere species richness.

Weighted Gene Co-expression Network Analysis (WGCNA) further validated this perspective at the community functional level. The MEbrown module, most strongly correlated with root morphological and physiological traits, enriched key genes including β-galactosidase (*lacZ*, K01190), α-galactosidase (*rafA*, K07407), and *L-fucokinase* (*fucK*, K00879). These genes participate in galactose metabolism and the pentose phosphate pathway, respectively: the former constitutes a core pathway for microbial carbon utilization, while the latter provides NADPH and carbon skeletons to cells under cold stress, thereby countering oxidative stress [91]. The marked enrichment of these potential key associated genes indicates that cold stress selected microbial functional groups possessing specialized carbon metabolism and energy supply strategies, thereby driving functional adaptation within the community. Previous studies have also confirmed that, in cold habitats such as Arctic permafrost, phyla with similar metabolic potential—including *Acidobacteria*, *Actinobacteria*, and *Chloroflexota*—maintain a high activity [92,93,94,95,96,97].

Consequently, the impact of low temperatures on rice rhizosphere microbial communities results from the combined processes of suppression and functional enrichment. The overall reduction in metabolic activity and nutrient fixation within the rice rhizosphere microbiome may lead to decreased community diversity. However, plant–microbial interactions (such as those driven by root exudates) and environmental selection (such as cold stress) jointly promote the functional enrichment of microbial groups (e.g., *Pseudomonadales*) harboring specific stress-resistant metabolic pathways (e.g., genes associated with the MEbrown module) [98,99]. The rhizosphere microbial communities of the two rice varieties exhibited differential responses to low temperatures in this study. DN428 stably enriched *Pseudomonadales* with specific functions, accompanied by alterations in the abundance of key functional genes (consistent with metagenomic data reflecting gene presence/abundance), whereas the significant shift in the diversity index of SJ10 further confirmed that this pathway of “functional remodeling” is variety-dependent. Ultimately, whether through the conservation of key functional genes (DN428) or the adjustment of broader community structure (SJ10), these cold-adaptive alterations in microbial communities profoundly influence their capacity to serve host plants—particularly in root development and nitrogen accumulation—thereby determining the overall cold tolerance of rice.

### 4.4. Integrative Model of Variety-Dependent Yield Loss Mechanisms Under Low-Temperature Treatment

The findings of this study indicate that low-temperature treatment reduces the final yield of rice varieties DN428 and SJ10 through distinct physiological and ecological pathways. Although the yield reduction induced by low temperatures shares common endpoints, root physiological inhibition and diminished nitrogen assimilation capacity, the upstream driving pathways exhibit fundamental divergence, reflecting the variety-specific nature of plant–microbe interaction patterns [100,101].

In the relatively cold-tolerant DN428, the yield reduction is associated with changes in the abundance of root-associated microbial functional genes (rather than a causal chain). Cold stress (particularly the T3 treatment) did not cause a significant collapse in its rhizosphere microbial α-diversity, but markedly decreased the abundance of key microbial functional genes associated with galactose metabolism (*lacZ*, *rafA*) and the pentose phosphate pathway (*fucK*). The decreased abundance of these genes is correlated with impaired microbial carbon utilization efficiency and core energy metabolism, which may weaken the community’s capacity to support plant stress resistance via antioxidant defense-related energy supply (such as NADPH) [102,103]. The immediate consequence was an inhibition of microbially mediated organic matter mineralization processes (particularly nitrogen transformation) [104], manifested as a decline in the relative abundance of core mineralizing functional groups such as the phylum *Actinomycetota*. This precipitated a sharp decline in the effective nitrogen supply to the rhizosphere, which in turn exerted dual signal and resource constraints. This indirectly yet powerfully suppressed root meristem activity, the function of nitrogen transporters (such as AMTs and NRTs), and the overall nitrogen uptake and assimilation efficiency [105,106,107,108].

In stark contrast, the yield reduction in the cold-sensitive cultivar SJ10 originates from a more macro-level “instability in microbial community structure”. Cold stress is associated with a significant reduction in its rhizosphere microbial α-diversity and a drastic restructuring of the community composition (e.g., reduced *Pseudomonadota* abundance, a phylum linked to carbon–nitrogen cycling), which is correlated with a loss of functional redundancy and degraded ecological functions [86]. For instance, disrupted organic acid metabolism affects soil pH and phosphorus availability, collectively leading to a comprehensive decline in beneficial rhizosphere ecological functions [109]. Against this backdrop, although SJ10 roots may attempt to initiate certain physiological stress responses, the sharp decline in bioavailable nutrients in the rhizosphere and the absence of beneficial microbial support collectively constitute powerful external constraints. These ultimately overwhelm the plant’s own physiological regulatory potential, resulting in a more severe direct suppression of root development and nitrogen uptake capacity.

In summary, this integrated model reveals that cold tolerance differences among rice varieties stem from a divergent reliance on distinct “plant–microbe” interaction axes. DN428 exhibits a “functionally robust” phenotype, relying on a core microbial community with stable functional gene expression. This buffers stress by maintaining key rhizosphere biogeochemical cycling functions, thereby securing space and resources for the plant’s own physiological regulation. Conversely, SJ10 exemplifies a “structurally fragile” response, wherein the structural vulnerability of its rhizosphere microbial community is amplified under stress. This leads to the premature collapse of ecosystem service functions, directly exposing plant physiological processes to the core stressors. Both pathways ultimately converge upon the shared bottleneck of root physiology and nitrogen metabolism. Yet their fundamental divergence provides a clear direction for variety-specific cold tolerance breeding and management: enhancing the functional resilience of the core microbiome in DN428-type varieties, versus fostering structurally stable microbial communities for SJ10-type varieties (Figure S10). Consequently, understanding crop stress resistance must shift from a singular organism-centric towards an integrated regulatory paradigm based on variety-specific “plant–microbe interaction modules”.

## 5. Conclusions

Low-temperature treatment significantly inhibited the root morphological and physiological traits of rice. Among them, the root length, root surface area, root volume, xylem sap rate, root oxidation activity, root nitrogen accumulation and biomass of the T3 treatment showed the greatest decline. It is worth noting that DN428 has a greater cold resistance, and its decline rate during each period was lower than that of SJ10. Low-temperature treatment changed the abundance and diversity of rhizosphere soil microorganisms and the abundance of related metabolic pathways: weighted gene co-expression network (WGCNA) analysis shows that *lacZ* (K01190), *fucK* (K00879) and *rafA* (K07407) in the MEbrown module are the key genes in response to the low-temperature treatment. Furthermore, a significant variety-dependent mechanism has been identified in the process of low-temperature treatment inducing a decrease in yield. During the period of rice growth that is sensitive to low temperatures, low-temperature treatment in the root zone can result in a decrease in the yield of rice varieties DN428 and SJ10 through differentiated physiological and ecological pathways. The yield decline in DN428 is predominantly associated with a decreased abundance of root-associated microbial functional genes, while that in SJ10 is primarily linked to the disrupted structure and function of its root-associated microbial community. However, both varieties ultimately exhibit inhibited root morphological and physiological traits and reduced nitrogen assimilation capacity, leading to a significant decrease in rice yield.

## Figures and Tables

**Table 1:** Effect of low-temperature treatment on yield components of *Oryza sativa* var. *japonica* in cold region during tiller and booting stage.

Year	Variety	Treatment	Effective Panicles (×10^4^ hm^−2^)	Number of Grains per Panicle	Seed Setting Rate (%)	1000-Grain Weight (g)	Yield (kg/hm^2^)
2023	DN428	T0	382.68 a	117.01 a	97.94 a	25.78 a	8871.41 a
		T1	363.66 b	108.43 b	92.79 b	25.18 a	7391.04 b
		T2	379.06 a	102.01 c	81.76 c	24.16 b	6083.13 c
		T3	345.68 c	93.96 d	71.65 d	23.03 c	4882.30 d
	SJ10	T0	370.21 a	112.01 a	96.76 a	24.21 a	8670.28 a
		T1	335.30 b	101.73 b	90.57 b	23.28 a	7056.12 b
		T2	365.22 a	96.01 c	78.00 c	22.09 b	5782.06 c
		T3	313.28 c	86.96 d	67.69 d	21.27 c	4692.51 d
2024	DN428	T0	380.40 a	116.67 a	97.62 a	25.34 a	8815.75 a
		T1	361.32 b	107.86 b	92.72 b	24.93 a	7338.44 b
		T2	377.17 a	101.67 c	81.22 c	23.88 b	6077.95 c
		T3	343.57 c	93.48 d	71.75 d	22.72 c	4850.21 d
	SJ10	T0	368.80 a	111.62 a	96.45 a	24.07 a	8611.52 a
		T1	332.75 b	101.43 b	90.15 b	22.97 a	7042.64 b
		T2	364.14 a	95.70 c	77.55 c	21.80 b	5739.95 c
		T3	310.52 c	86.61 d	68.16 d	20.95 c	4655.67 d
			F-value				
		Y	0.830	2.645	2.460	3.161	3.688
		T	95.060 **	1833.364 **	3566.571 **	57.759 **	7821.107 **
		V	92.482 **	669.796 **	201.938 **	118.995 **	177.649 **
		Y*T	0.011	0.011	0.098	0.003	0.136
		Y*V	0.002	0.040	0.485	0.034	0.001
		T*V	5.403 **	3.088 *	11.651 **	0.738	3.232 *
		Y*T*V	0.007	0.021	0.851	0.061	0.163

Note: Values within a column followed by different letters are significantly different at *p* < 0.05. * Significant at *p* < 0.05. ** Significant at *p* < 0.01.

**Table 2:** Analysis of Alpha diversity. Values with a column followed by different letters are significantly different at *p* < 0.05. * Significant at *p* < 0.05; ** Significant at *p* < 0.01.

Growth Stage	Variety	Treatment	Ace	Shannon	Simpson
Tillering	DN428	CK1	38,684 a	7.10 b	0.0062 a
		T1	38,281 a	7.12 a	0.0061 a
	SJ10	CK1	38,368 b	7.10 a	0.0064 a
		T1	38,910 a	7.08 a	0.0062 a
	F-value	T	0.364	2.414	3.433
		V	1.852	14.740 **	5.798 *
		T*V	16.835 **	15.643 **	0.151
Meiosis	DN428	CK2	37,960 a	7.12 a	0.0057 a
		T2	38,106 a	7.11 a	0.0059 a
		T3	38,429 a	7.12 a	0.0057 a
	SJ10	CK2	38,395 a	7.11 a	0.0060 a
		T2	38,394 a	7.12 a	0.0057 b
		T3	38,492 a	7.08 b	0.0061 a
	F-value	T	0.737	2.556	1.805
		V	1.753	3.092	3.823
		T*V	0.300	5.087 *	6.203 *
	DN428	CK3	38,061 a	7.08 b	0.0062 a
		T1	38,128 a	7.10 a	0.0059 b
Full heading		T2	38,165 a	7.12 a	0.0060 b
		T3	38,033 a	7.12 a	0.0061 ab
	SJ10	CK3	38,441 a	7.10 a	0.0060 b
		T1	38,325 a	7.06 b	0.0064 a
		T2	37,597 b	7.11 a	0.0056 c
		T3	38,165 a	7.09 a	0.0062 b
	F-value	T	3.019	6.516 **	11.123 **
		V	0.131	7.062 *	0.020
		T*V	4.551 *	6.267 **	18.466 **
Mature	DN428	CK4	38,064 a	7.10 ab	0.0058 a
		T1	38,215 a	7.11 a	0.0058 a
		T2	38,006 a	7.07 b	0.0059 a
		T3	38,600 a	7.10 ab	0.0060 a
	SJ10	CK4	38,798 a	7.10 a	0.0060 ab
		T1	38,393 b	7.09 a	0.0061 ab
		T2	38,483 ab	7.10 a	0.0058 b
		T3	38,649 ab	7.08 a	0.0061 a
	F-value	T	2.126	1.134	1.772
		V	9.707 **	0.193	3.931
		T*V	1.780	3.212	0.699

## Data Availability

The metagenomic sequencing data reported in this study have been deposited in the Sequence Read Archive (SRA) database under the accession number of PRJNA1397335.

## Supplementary Materials

The following supporting information can be downloaded at: https://www.mdpi.com/article/10.3390/microorganisms14030632/s1, Table S1: Specific handling method. Table S2: Effect of low-temperature treatment on root morphological and physiological traits, root nitrogen accumulation and biomass of japonica rice in cold region during tillering and booting stage. Table S3: Three-way analysis of variance (*p* values) for linking microbial features to plant and soil parameters. Table S4: Three-way analysis of variance (*p* values) for microbial phyla correlated with soil nutrients. Figure S1: Box plots showing the cold-reaction index (CRI) of root surface area (a,b) and root volume (c,d) of rice varieties Songjing 10 (SJ10) and Dongnong 428 (DN428) under low-temperature treatment at the tillering and booting stage during 2023 and 2024. Figure S2: Community composition analysis of rhizosphere soil microorganisms at tillering stage (a), meiosis (b), full heading (c) and mature stages (d) at the phylum level under low-temperature treatment at the tillering and booting stages. D is DN428; S is SJ10. CK1–CK4 are the corresponding control group data for the tillering stage, meiosis stage, full heading stage and mature stage, respectively. Figure S3: Heatmap of rhizosphere soil microorganisms at tillering stage (a), meiosis (b), full heading (c) and maturity stages (d) at the phylum level under low-temperature treatment at the tillering and booting stages. D is DN428; S is SJ10. CK1–CK4 are the corresponding control group data for the tillering stage, meiosis stage, full heading stage and mature stage, respectively. Figure S4: RDA of rhizosphere soil microorganisms at tillering stage (a), meiosis (b), full heading (c) and maturity stages (d) at the phylum level under low-temperature treatment at the tillering and booting stages. D is DN428; S is SJ10. CK1–CK4 are the corresponding control group data for the tillering stage, meiosis stage, full heading stage and mature stage, respectively. Figure S5: Correlation heatmap analysis between rhizosphere soil microorganisms and soil pH and nutrient contents; D is DN428; S is SJ10; T is the corresponding treatment group; CK is the control group at phylum level under low-temperature treatment at tillering and booting stages. Figure S6: Relative abundance of rhizosphere soil microbial communities at the phylum level under low-temperature treatments. D is DN428; S is SJ10. CK1–CK4 are the corresponding control group data for the tillering stage (a), meiosis stage (b), full heading stage (c) and mature stage (d), respectively. Figure S7: Clustering tree and module division of genes for root traits. Figure S8: Clustering tree and module division of genes for root traits for soil nutrients. Figure S9: Correlation analysis between co-expression network module and soil pH and nutrient contents (a) (SOM: soil organic matter; TN: total nitrogen; TP: total phosphorus; AK: available potassium; AP: available phosphorus). Regulatory network of key genes in the MEblue module (b). Regulatory network of key genes in the MEgreen module (c). Figure S10: Article flowchart.

## Abbreviations

The following abbreviations are used in this manuscript:

RL	Root length
RV	Root volume
RS	Root surface area
XSR	Xylem sap rate
ROA	Root oxidation activity
SOM	Soil organic matter
TN	Total nitrogen
TP	Total phosphorus
AP	Available phosphorus
AK	Available potassium
