# Supplementary material for: Low Temperature Impacts Root Physiological Characteristics and Related Microbial Community Diversity in the Rhizosphere of Japonica Rice
Source: Microorganisms. 2026 Mar 11;14(3):632. doi: 10.3390/microorganisms14030632 (PMC13028632; doi:10.3390/microorganisms14030632)
Supplement: Supplementary file 1 [file microorganisms-14-00632-s001.zip › microorganisms-4116653-supplementary.pdf]

Table S1. Specific handling method

Treatment	Processing mode
Room temperature control(CK)	The normal temperature control reference is the temperature in the sunken water pool.
Low temperaure at tillering stage(T1)	Starting from 7 days after the rice resumes growth, the cold water irrigation treatment for the tillering stage was carried out. The treatment lasted for 7 days, and the temperature of the irrigation water was 15±0.5℃.
Low temperaure at booting stage(T2)	When the spikelets reach a length of about 1 cm, start the cold water irrigation treatment during the booting stage. The treatment lasts for 7 days, and the temperature of the water is 17±0.5℃.
Mixed low temperaure at tillering and booting stage(T3)	Cold water irrigation was applied during both the tillering and booting stage of rice growth. The cumulative treatment duration was 14 days. The irrigation water temperature was 15±0.5℃ during the tillering stage and 17±0.5℃ during the booting stage.

Table S2. Effect of low-temperature treatment on root morphological and physiological traits, root nitrogen accumulation and biomass of japonica rice in cold region during tillering and booting stage

	F	Growth stage							
	F-value	Tillering	Jointing	Meiosis	Full heading	Grouting	Milky	Dough	Mature
Root length	Y	1.070	0.883	0.179	0.191	0.468	0.264	1.223	0.705
	T	337.917**	237.144**	596.098**	506.628**	465.058**	454.190**	740.836**	497.471**
	V	19.352**	10.469**	90.129**	54.570**	103.228**	33.699*	342.094**	191.077**
	Y*T	0.063	0.014	0.092	0.060	0.123	0.127	0.012	0.012
	Y*V	0.073	0.039	0.009	0.007	0.067	0.186	0.001	0.014
	T*V	0.038	0.002	0.173	0.289	0.038	0.466	0.009	0.005
	Y*T*V	0.005	0.007	0.019	0.035	0.049	0.033	0.017	0.005
Root surface	Y	0.372	0.281	0.605	0.509	1.230	0.051	0.011	0.000
	T	1974.935**	495.978**	1212.938**	1389.658**	1246.800**	1356.110**	1727.692**	1921.325**
	V	164.455**	139.448**	133.473**	95.856**	138.215**	26.772**	16.150**	135.226**
	Y*T	0.000	0.129	0.950	0.700	0.266	0.384	0.369	0.201
	Y*V	0.001	0.094	0.018	0.002	0.182	0.685	0.184	0.000
	T*V	0.038	0.270	0.042	0.354	0.221	0.087	0.087	0.295
	Y*T*V	0.010	0.215	0.060	0.100	0.156	0.100	0.019	0.191
Root volume	Y	3.741	1.103	3.694	2.775	1.974	3.396	2.915	1.181
	T	102.029**	1006.267**	2739.340**	4324.210**	2152.340**	4485.339**	1512.160**	1989.312**
	V	55.295**	21.023**	318.287**	781.613**	251.462**	416.008**	296.102**	146.188**
	Y*T	0.454	0.011	11.804**	2.183	0.982	0.224	3.478*	3.381*
	Y*V	0.218	0.011	0.451	0.369	27.458**	12.173**	7.905**	0.115
	T*V	0.158	0.454	0.189	3.543*	0.736	1.301	0.890	3.876*
	Y*T*V	0.030	0.003	0.046	0.502	0.547	0.293	0.394	2.779
Xylem sap rates	Y	3.556	0.196	0.501	0.000	0.867	0.128	1.148	0.088
	T	187.773**	264.355**	490.084**	620.043**	881.588**	361.821**	429.792**	297.086**
	V	91.370**	277.587**	51.504**	103.432**	120.700**	62.118**	119.760**	69.274**
	Y*T	0.035	0.587	0.072	0.090	0.005	0.176	0.488	0.027
	Y*V	0.004	1.761	0.255	1.249	0.234	0.357	0.094	0.573
	T*V	0.509	0.065	0.535	0.431	0.745	0.266	0.119	0.047
	Y*T*V	0.069	0.181	0.140	0.027	0.053	0.043	0.098	0.108

Root oxidation activity	Y	1.852	1.270	0.287	0.288	0.003	0.685	0.073	0.363
	T	2196.772**	1089.009**	2216.375**	2417.383**	2159.049**	1569.288**	1360.871**	792.037**
	V	221.153**	0.407	225.427**	240.858**	329.746**	77.000**	284.284**	64.319**
	Y*T	0.000	0.690	0.527	0.019	0.260	0.176	0.325	0.284
	Y*V	0.051	2.501	4.324*	25.886**	5.915*	12.990**	1.422	2.477
	T*V	0.186	31.491**	0.722	0.682	0.053	2.041	1.813	1.336
	Y*T*V	0.008	0.418	0.253	0.545	0.077	0.632	0.512	0.433
Root nitrogen accumulation	Y	0.166	0.919	1.509	0.097	0.414	0.052	0.066	0.212
	T	235.400**	257.514**	329.355**	868.115**	993.368**	448.367**	324.889**	350.003**
	V	73.691**	170.885**	105.680**	84.798**	408.679**	211.681**	266.132**	409.374**
	Y*T	1.421	0.208	0.756	0.365	1.853	0.764	0.685	0.367
	Y*V	0.027	1.181	0.008	0.320	0.399	0.002	0.049	0.063
	T*V	0.949	0.812	0.101	1.670	0.136	0.446	1.578	0.835
	Y*T*V	0.061	0.401	0.056	0.121	0.195	0.067	0.012	0.162
Biomass	Y	0.229	0.238	0.034	0.000	2.603	1.080	0.377	0.788
	T	338.420**	476.041**	1205.789**	2226.683**	2627.420**	1573.752**	1946.750**	1951.968**
	V	119.684**	364.505**	92.355**	231.933**	440.887**	293.930**	363.737**	201.948**
	Y*T	0.017	0.033	1.284	1.283	0.374	0.644	2.140	5.669**
	Y*V	0.123	0.143	0.001	0.080	1.170	0.466	0.022	0.374
	T*V	0.041	0.477	0.327	3.311*	3.534*	2.117	3.163*	3.888*
	Y*T*V	0.122	0.036	0.019	0.023	0.564	0.315	0.570	0.300

Table S3. Three-way analysis of variance (p values) for linking microbial features to plant and soil parameters

Index	Root length	Root surface area	Root volume	Xylem sap rate	Root oxidation activity	AK	AP	TN	TP
Actinomycetota	<0.001	<0.001	<0.001	<0.001	<0.001	<0.001	<0.001	0.015	<0.001
Euryarchaeota	0.015	0.023	0.029	0.031	0.134	0.003	0.001	0.371	0.037
Thermodesulfobacteriota	0.002	<0.001	<0.001	<0.001	<0.001	0.001	<0.001	0.015	0.003
Chloroflexota	0.080	0.087	0.275	0.191	0.455	0.012	0.006	0.813	0.100

Table S4. Three-way analysis of variance (p values) for microbial phyla correlated with soil nutrients

Group	Index	SOM	TN	TP	AK	AP
DN428 treatment	Pseudomonadota	0.632	0.003	0.004	0.906	0.009
	Cyanobacteriota	0.934	0.009	0.006	0.188	0.443
	Acidobacteriota	0.337	0.054	0.144	0.065	0.003
	Actinomycetota	0.007	0.115	0.031	0.440	0.002
	Bacteroidota	0.723	0.071	0.054	<0.001	0.226
	Thermodesulfobacteriota	<0.001	0.185	0.121	0.429	0.528
DN428 control	Pseudomonadota	0.059	0.042	0.005	0.430	0.007
	Acidobacteriota	0.059	0.006	<0.001	0.297	<0.001
	Actinomycetota	0.015	0.028	0.002	0.308	0.004
	Gemmatimonadota	0.059	0.006	<0.001	0.297	<0.001
SJ10 treatment	Myxococcota	0.048	0.159	0.080	0.008	0.265
	Gemmatimonadota	0.068	<0.001	<0.001	0.825	0.161
	Candidatus Rokubacteria	0.107	0.346	0.401	0.573	<0.001
SJ10 control	Gemmatimonadota	0.010	0.029	0.053	0.297	0.003
	Candidatus Rokubacteria	0.152	0.004	0.073	0.033	0.006
	Actinomycetota	0.002	0.378	0.097	0.746	0.118
	Verrucomicrobiota	0.255	<0.001	<0.001	0.090	<0.001

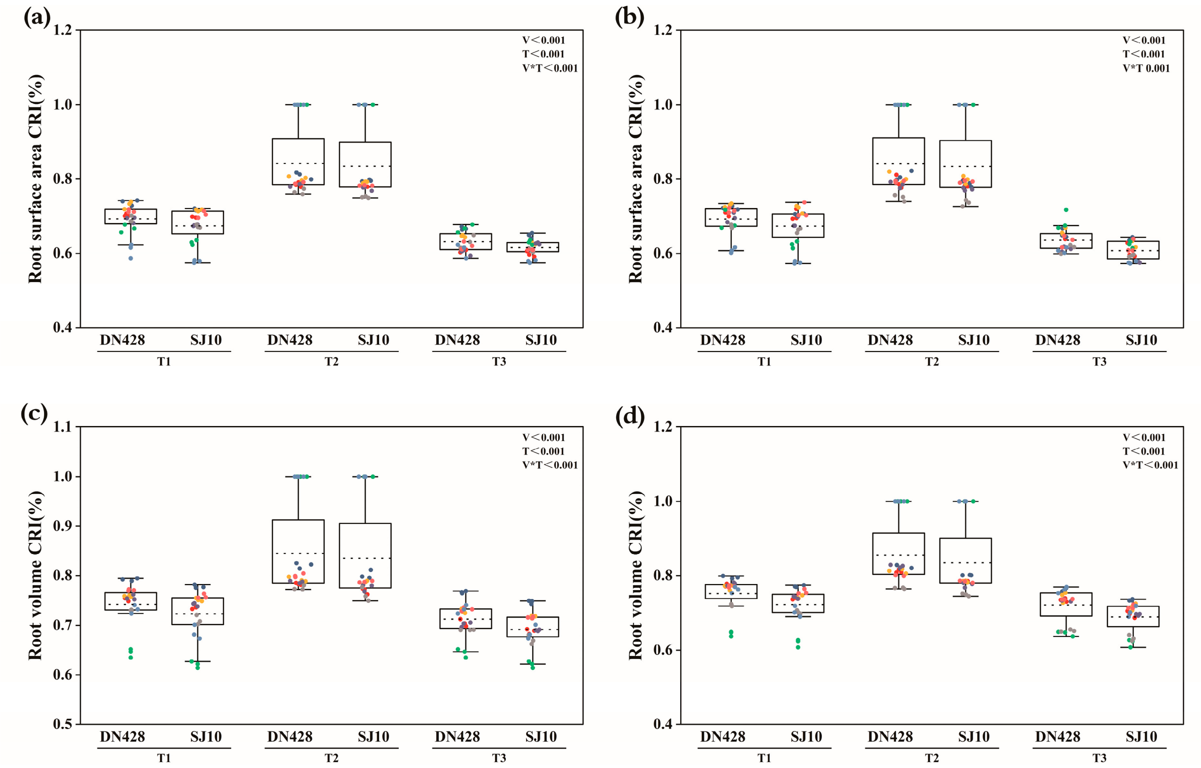

Figure S1. Box plots showing the cold-reaction index (CRI) of root surface area(**a-b**) and root volume (**c-d**) of rice varieties Songjing 10(SJ10) and Dongnong 428(DN428) under low-temperature treatment at the tillering and booting stage during 2023 and 2024. The significant levels of variety (V) and temperature(T) effects and their interactions are also shown. Data from different growth stages are pooled.

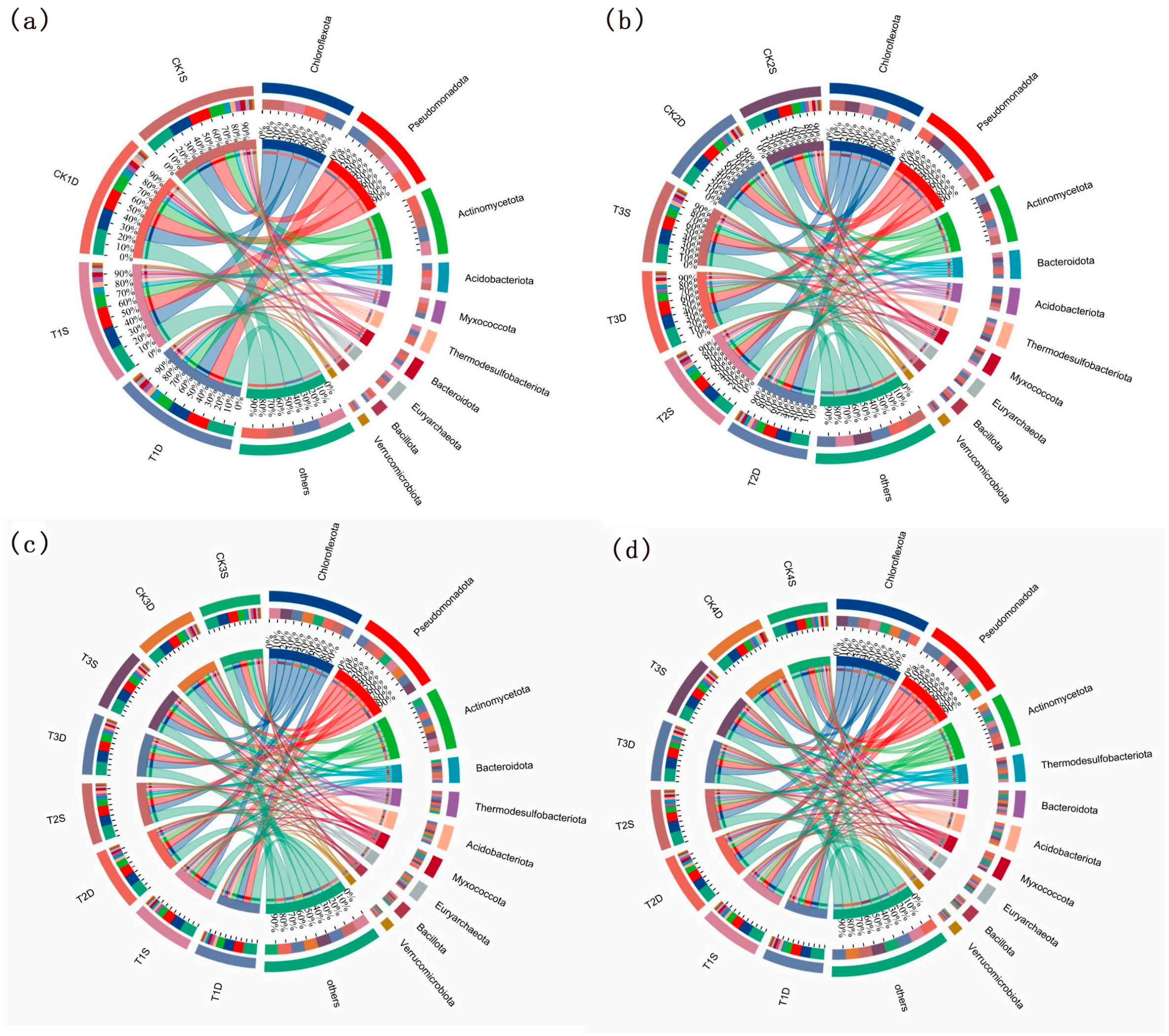

Figure S2. Community composition analysis of rhizosphere soil microorganisms at tillering stage (**a**),meiosis(**b**),full heading(**c**) and mature stages(**d**)at the phylum level under low-temperature treatment at the tillering and booting stages.D is DN428,S is SJ10.CK1-CK4 are the corresponding control group data for the tillering stage, meiosis stage, full heading stage and mature stage respectively.

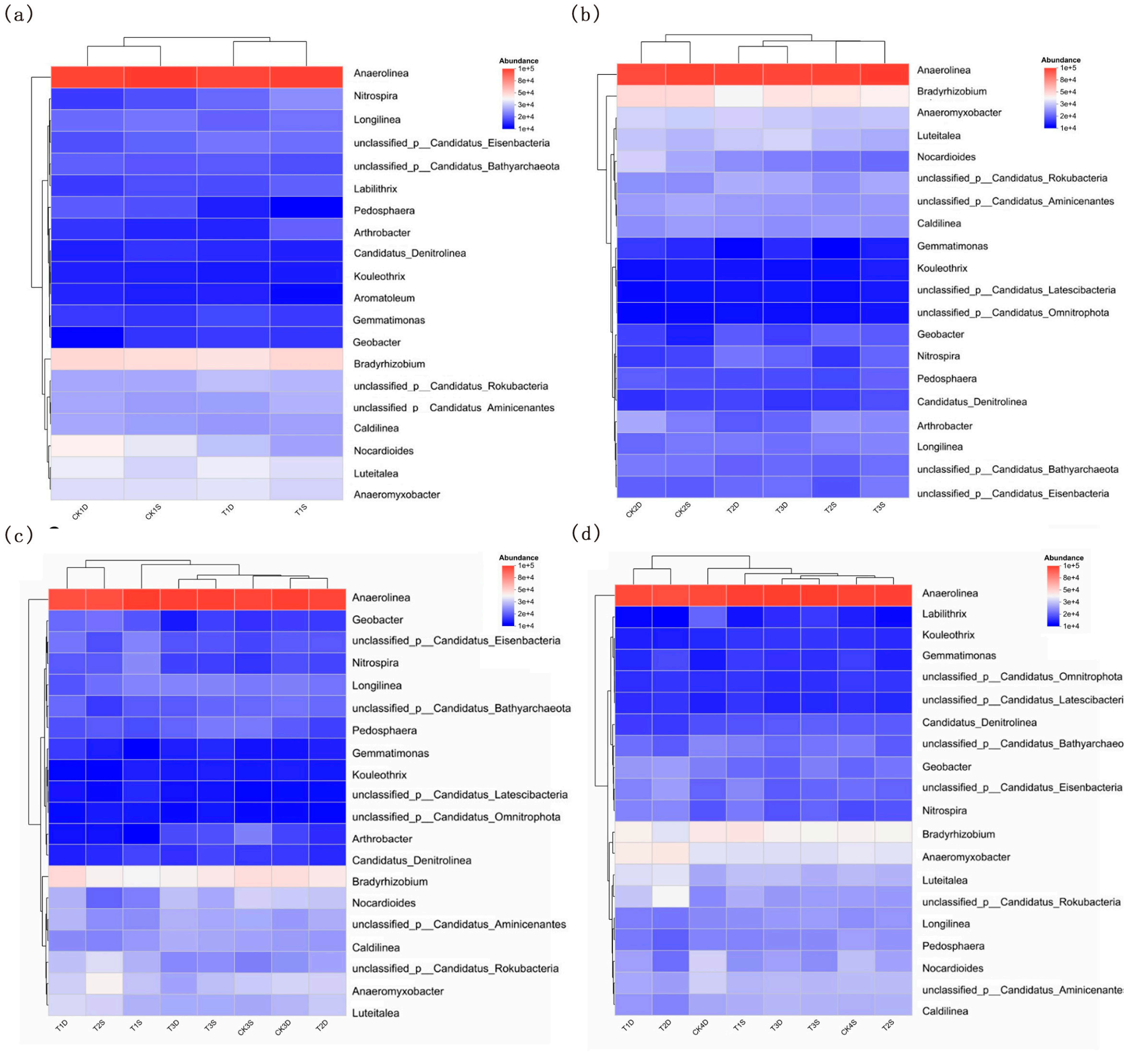

Figure S3. Heatmap of rhizosphere soil microorganisms at tillering stage (**a**), meiosis (**b**), full heading (**c**) and maturity stages (**d**) at the phylum level under low-temperature treatment at the tillering and booting stages.D is DN428,S is SJ10.CK1-CK4 are the corresponding control group data for the tillering stage, meiosis stage, full heading stage and mature stage respectively

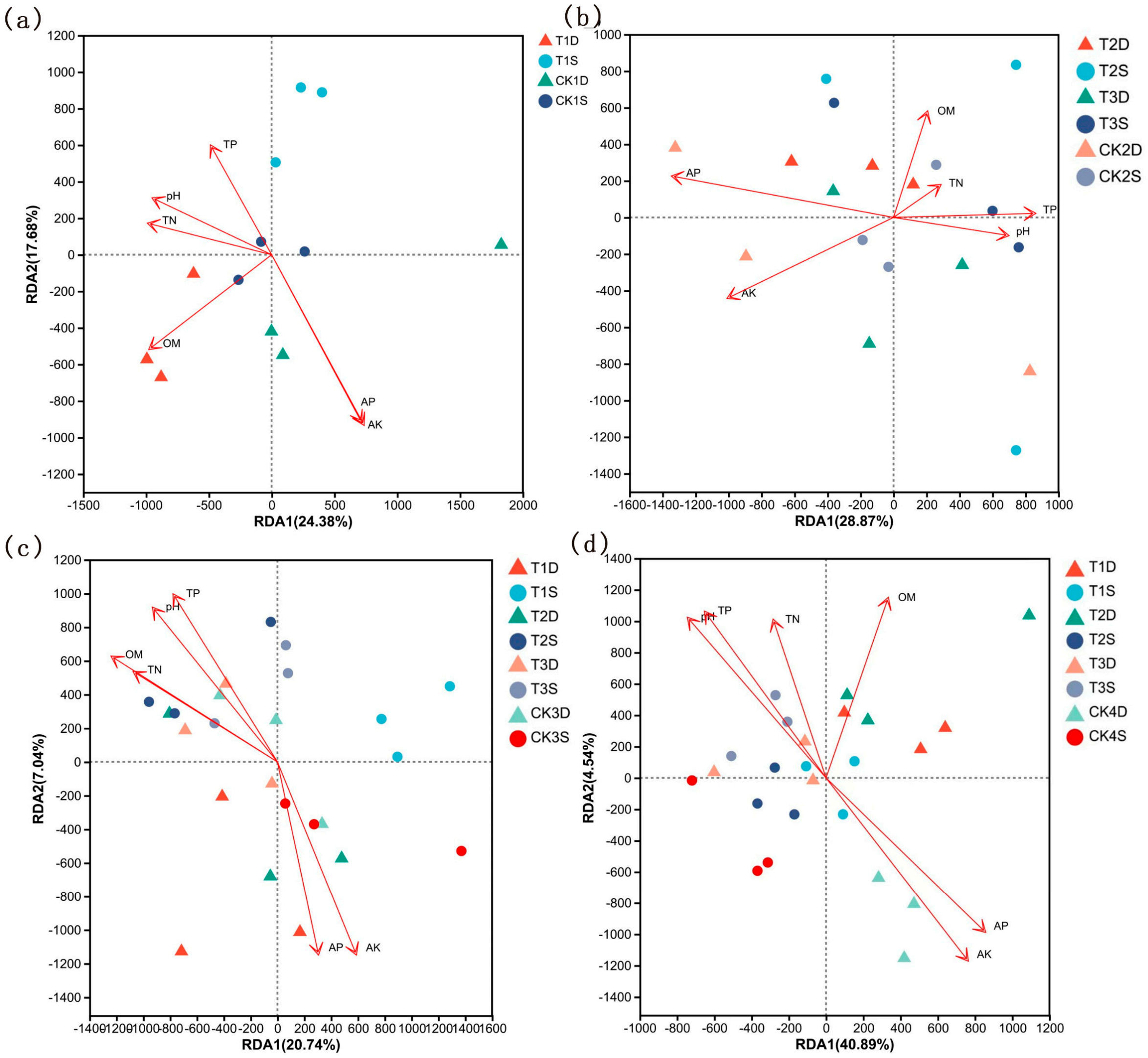

Figure S4. RDA analysis of rhizosphere soil microorganisms at tillering stage (**a**), meiosis (**b**), full heading (**c**) and maturity stages (**d**) at the phylum level under low-temperature treatment at the tillering and booting stages. D is DN428,S is SJ10.CK1-CK4 are the corresponding control group data for the tillering stage, meiosis stage, full heading stage and mature stage respectively.

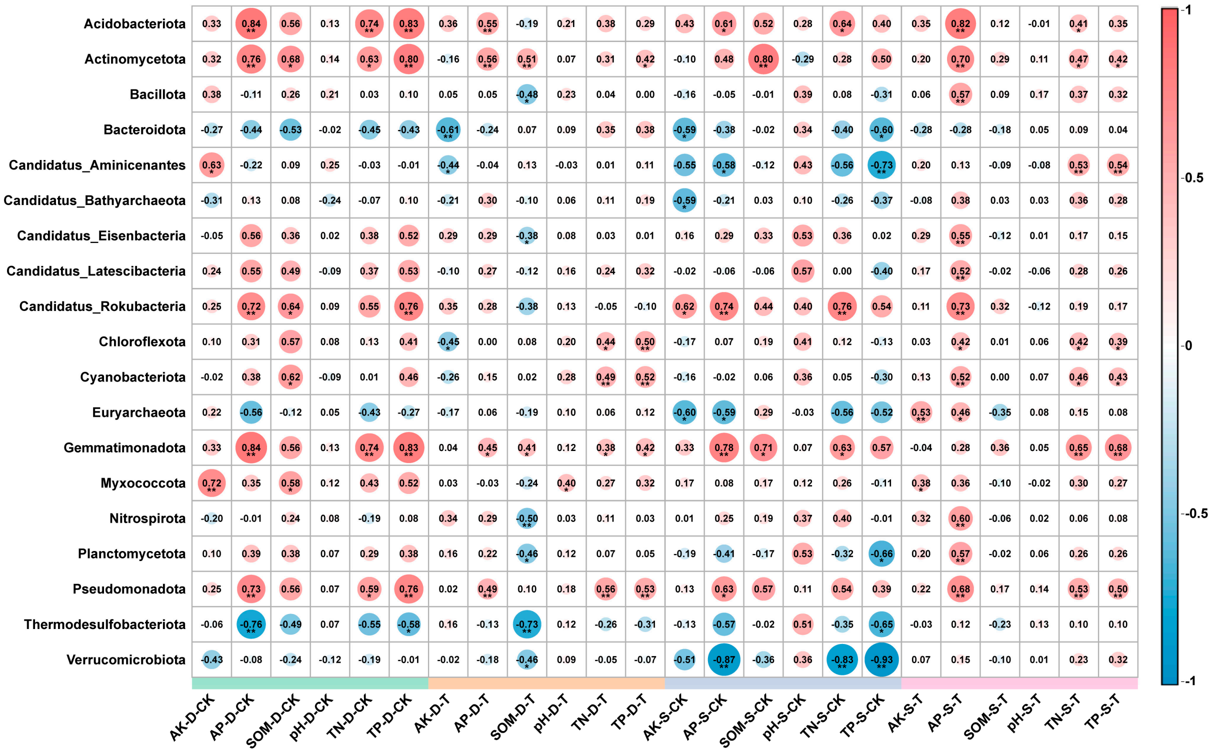

Figure S5. Correlation heatmap analysis between rhizosphere soil microorganisms and soil pH and nutrient contents, D is DN428, S is SJ10, T is the corresponding treatment group, CK is the control group at phylum level under low-temperature treatment at tillering and booting stages. P value is displayed with asterisk.* represents P<0.05, ** represents P<0.01, *** represents P<0.001.

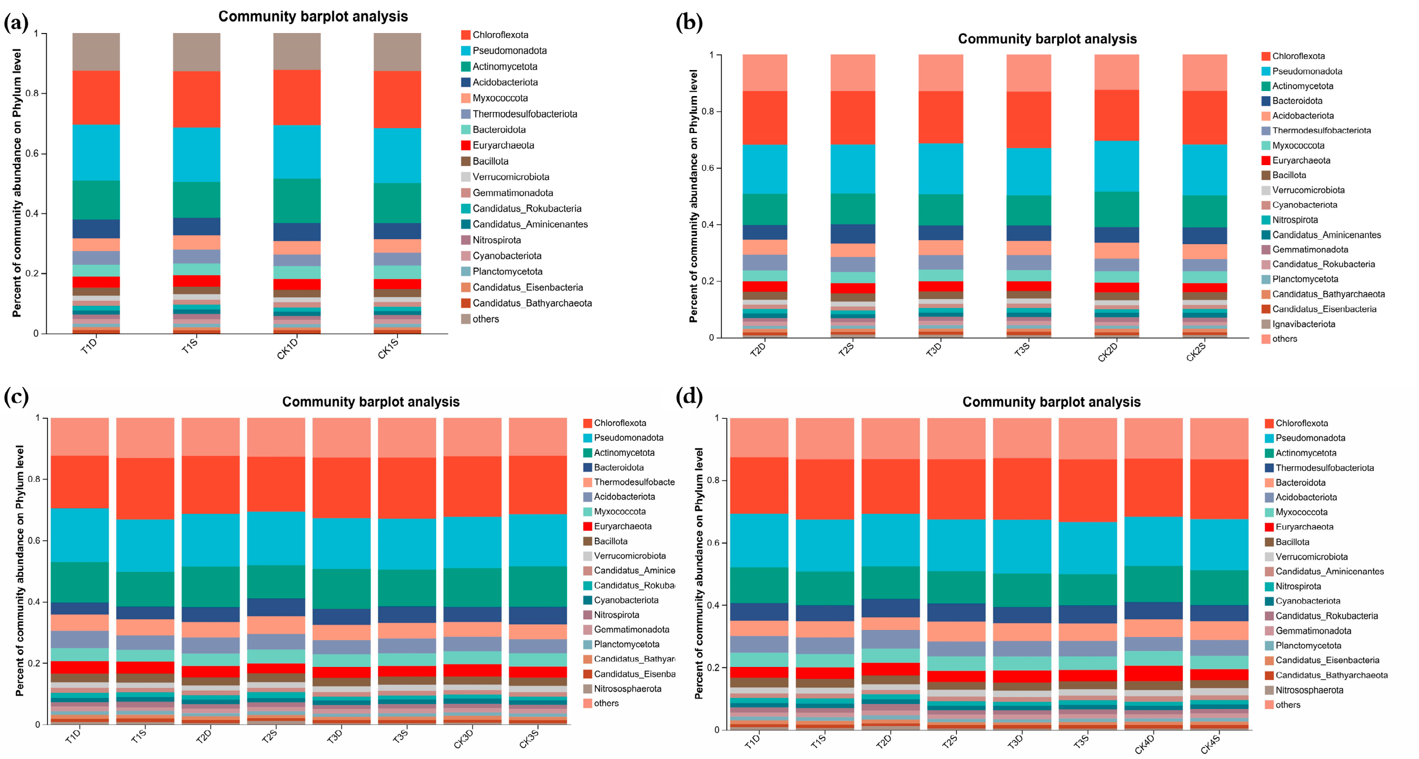

Figure S6. Relative abundance of rhizosphere soil microbial communities at the phylum level under low-temperature treatments.D is DN428,S is SJ10.CK1-CK4 are the corresponding control group data for the tillering stage(**a**), meiosis stage(**b**), full heading stage (**c**) and mature stage(**d**) respectively.

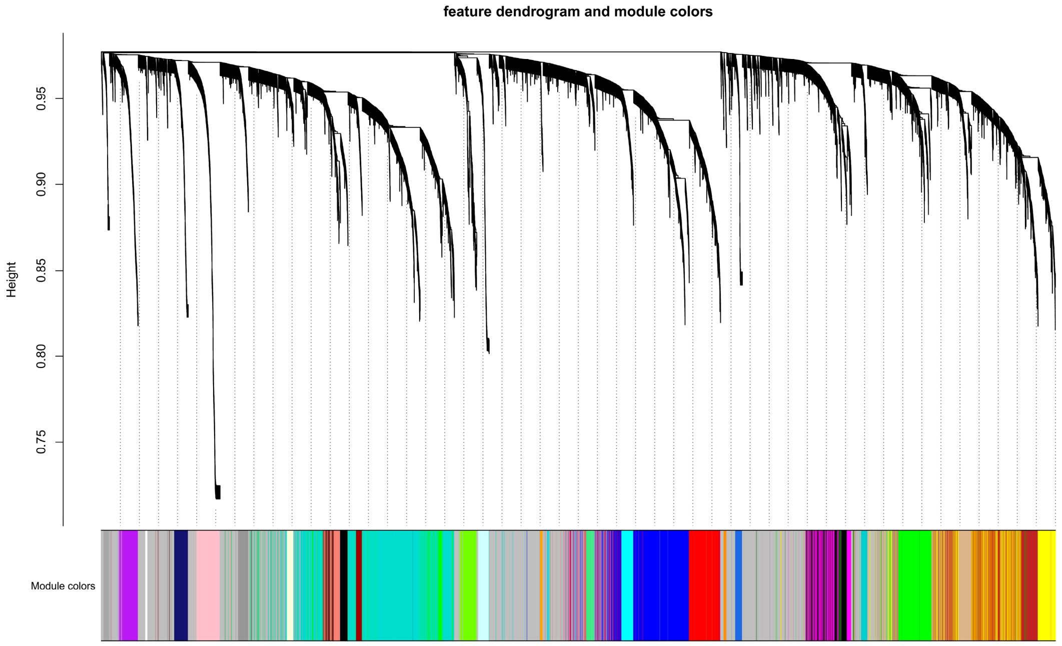

Figure S7. Clustering tree and module division of genes for root traits

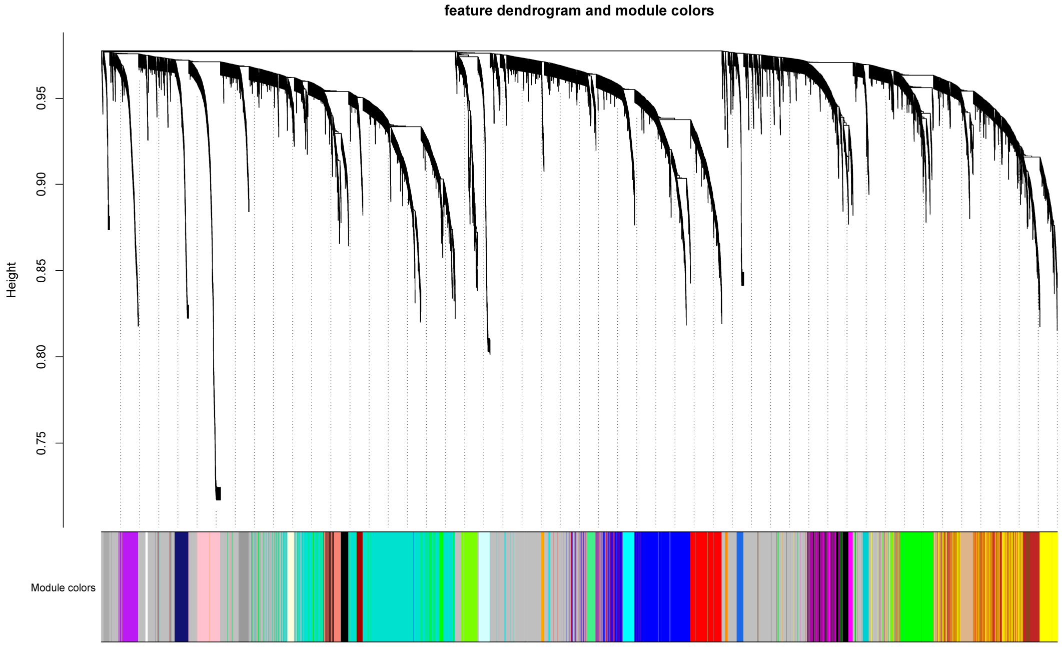

Figure S8. Clustering tree and module division of genes for root traits for soil nutrients

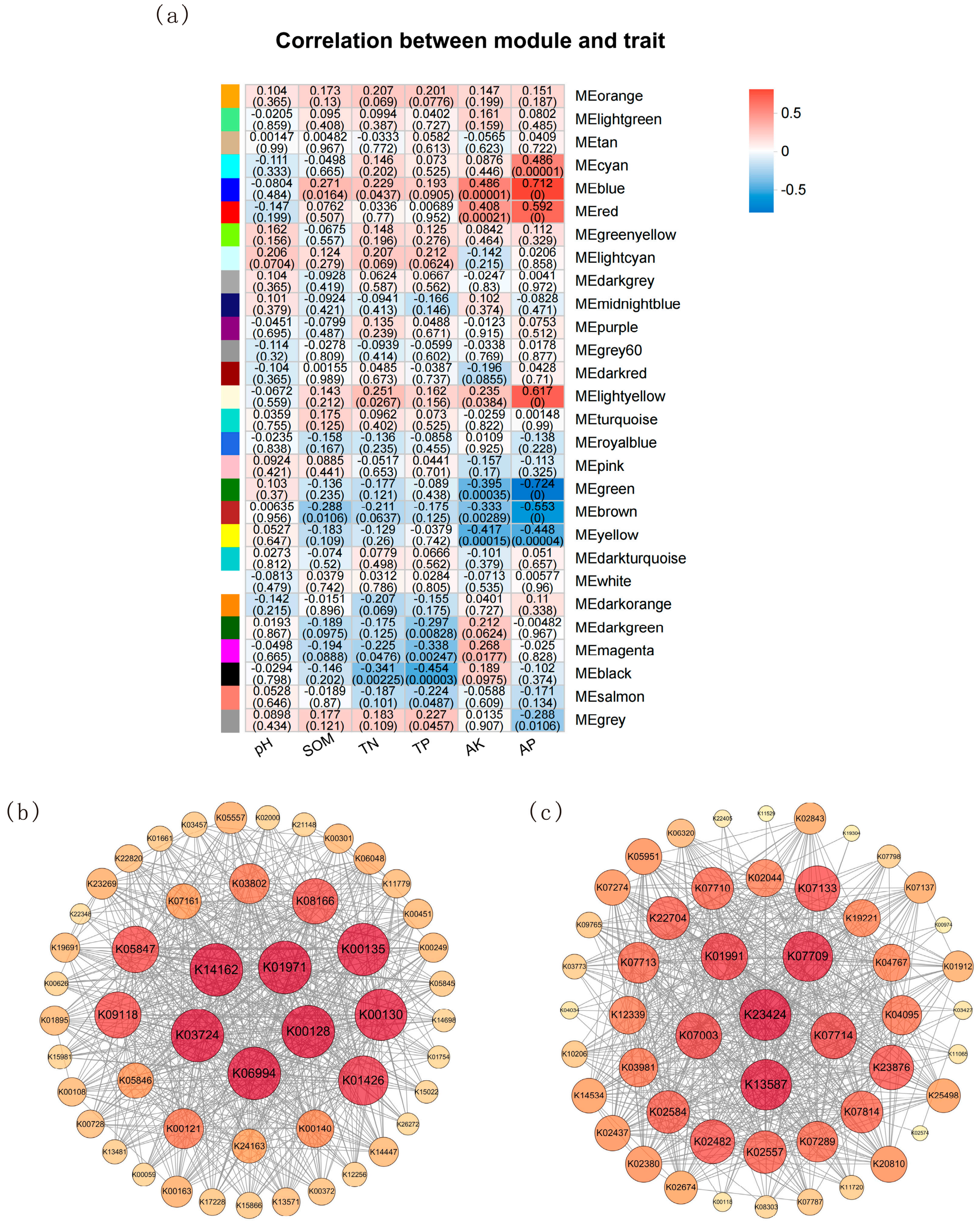

Figure S9. Correlation analysis between coexpression network module and soil pH and nutrient contents (**a**). (SOM: Soil organic matter; TN: Total nitrogen; TP: Total phosphorus; AK: Available potassium; AP: Available phosphorus). Regulatory network of key genes in the MEblue module (**b**). Regulatory network of key genes in the MEgreen module (**c**).

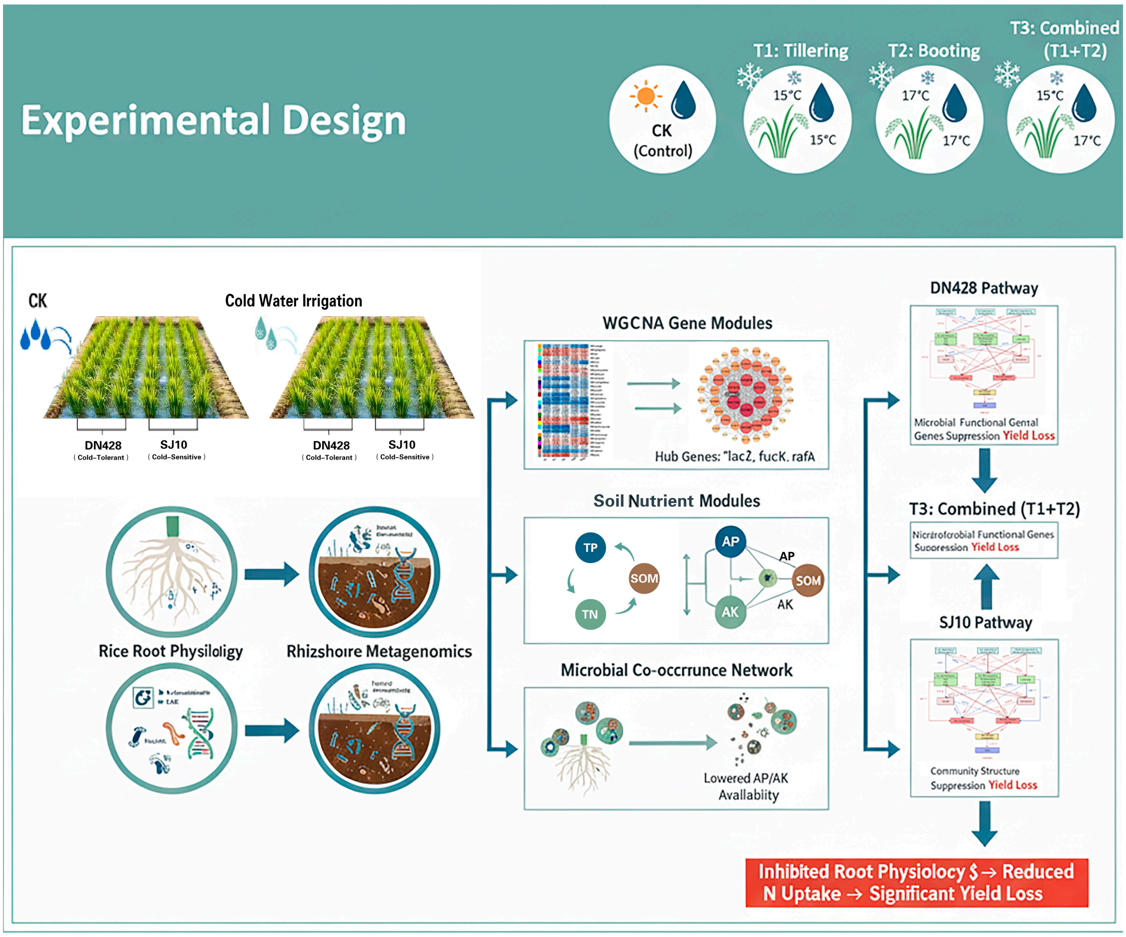

Figure S10. Article flowchart.
